# Endothelial Klf2-Foxp1-TGFβ signal mediates the inhibitory effects of simvastatin on maladaptive cardiac remodeling

**DOI:** 10.7150/thno.48153

**Published:** 2021-01-01

**Authors:** Hongda Li, Yanfang Wang, Jiwen Liu, Xiaoli Chen, Yunhao Duan, Xiaoyu Wang, Yajing Shen, Yashu Kuang, Tao Zhuang, Brain Tomlinson, Paul Chan, Zuoren Yu, Yu Cheng, Lin Zhang, Zhongmin Liu, Yuzhen Zhang, Zhenlin Zhao, Qi Zhang, Jie Liu

**Affiliations:** 1 Key Laboratory of Arrhythmias of the Ministry of Education of China, Research Center for Translational Medicine, Shanghai East Hospital, Tongji University School of Medicine, Shanghai, China.; 2 Department of Cardiology, Shanghai East Hospital, Tongji University School of Medicine, Shanghai, China.; 3 Institute for Biomedical Engineering and Nano Science, Tongji University School of Medicine, Shanghai, China.; 4 Faculty of Medicine, Macau University of Science and Technology, Macau SAR, China.; 5 Division of Cardiology, Department of Internal Medicine, Wan Fang Hospital, Taipei Medical University, Taipei, Taiwan.; 6 Shenzhen Ruipuxun Academy for Stem Cell and Regenerative Medicine, Shenzhen, China.

**Keywords:** Heart failure (HF), Maladaptive cardiac remodeling (cardiac fibrosis and hypertrophy), HMG-CoA reductase inhibitors, Simvastatin, Vascular endothelial cells (ECs), Krüppel-like Factor 2 (Klf2), Transforming growth factor-beta 1 (TGFβ1), Forkhead Box P1 (Foxp1)

## Abstract

**Aims:** Pathological cardiac fibrosis and hypertrophy are common features of left ventricular remodeling that often progress to heart failure (HF). Endothelial cells (ECs) are the most abundant non-myocyte cells in adult mouse heart. Simvastatin, a strong inducer of Krüppel-like Factor 2 (Klf2) in ECs, ameliorates pressure overload induced maladaptive cardiac remodeling and dysfunction. This study aims to explore the detailed molecular mechanisms of the anti-remodeling effects of simvastatin.

**Methods and Results:** RGD-magnetic-nanoparticles were used to endothelial specific delivery of siRNA and we found absence of simvastatin's protective effect on pressure overload induced maladaptive cardiac remodeling and dysfunction after *in vivo* inhibition of EC-Klf2. Mechanism studies showed that EC-Klf2 inhibition reversed the simvastatin-mediated reduction of fibroblast proliferation and myofibroblast formation, as well as cardiomyocyte size and cardiac hypertrophic genes, which suggested that EC-Klf2 might mediate the anti-fibrotic and anti-hypertrophy effects of simvastatin. Similar effects were observed after Klf2 inhibition in cultured ECs. Moreover, Klf2 regulated its direct target gene TGFβ1 in ECs and mediated the protective effects of simvastatin, and inhibition of EC-Klf2 increased the expression of EC-TGFβ1 leading to simvastatin losing its protective effects. Also, EC-Klf2 was found to regulate EC-Foxp1 and loss of EC-Foxp1 attenuated the protective effects of simvastatin similar to EC-Klf2 inhibition.

**Conclusions:** We conclude that cardiac microvasculature ECs are important in the modulation of pressure overload induced maladaptive cardiac remodeling and dysfunction, and the endothelial Klf2-TGFβ1 or Klf2-Foxp1-TGFβ1 pathway mediates the preventive effects of simvastatin. This study demonstrates a novel mechanism of the non-cholesterol lowering effects of simvastatin for HF prevention.

## Introduction

Heart failure (HF) is a condition from various forms of cardiovascular disease that is characterized by debilitating symptoms with a particularly poor quality of life, frequent hospital admissions and reduced survival in people over the age of 65 years 1, 2. Despite the improvements in therapeutic strategies for cardiovascular diseases 3, HF remains a leading cause of death worldwide indicating that additional therapies which reduce the risk of developing HF are highly desirable.

Coronary artery disease and its major risk factors, including hypertension, diabetes mellitus and dyslipidemia, are the common cause of end-stage HF. Because of this, the plasma cholesterol-lowering medication 3-Hydroxy-3-methylglutaryl coenzyme A (HMG-CoA) reductase inhibitors or statins, are often prescribed that not only reduce the cardiovascular-related morbidity and mortality, but they also reduce the HF incident and modestly reduce the hospitalization due to non-fatal HF. However, the benefit of statin therapy is more controversial once HF has developed 4, 5.

Interstitial fibrosis, chamber remodeling, and reduced ventricular compliance are the characterized features of HF 6, 7. Cardiac fibrosis is an integral constituent of every form of chronic heart disease and leads to an increased wall stiffness and diastolic dysfunction 8. Left ventricular (LV) hypertrophy occurs as an adaptive response to increased workload, but prolonged LV hypertrophy results in cardiac dysfunction eventually leading to HF and death 9-11. Conceptually, statin therapy could benefit patients with HF by multiple mechanisms and the effects independent of cholesterol-lowering are regarded as the pleiotropic effects of statins 12.

An early study published in *Circulation* demonstrated that HMG-CoA reductase inhibitor, simvastatin, was able to prevent LV hypertrophy and to slow the transition to HF in the murine transverse aortic constriction (TAC) model through inhibition of *in vivo ras* signaling in the heart 13. A more recent study showed that a small GTP-binding protein, GDP dissociation stimulator (SmgGDS), played crucial roles in the inhibitory effects of statins on cardiac hypertrophy and fibrosis, partly through the regulation of Rac1, Rho kinase and extracellular signal-regulated kinase 1/2 pathways, demonstrating both intracellular and extracellular effects of SmgGDS 14.

The heart is an organ consisting of different cell types, including myocytes, endothelial cells, fibroblasts, stem cells, and inflammatory cells. This pluricellularity provides the opportunity of intercellular communication within the organ, with subsequent optimization of the modulatory role in normal and failing heart of adults. Microvasculature endothelial cells (ECs) are reported to represent the major non-myocyte cell population in the total number in the adult mouse heart 15. Besides their structural role in the interior surfaces of blood vessels, vascular ECs are metabolically active and secrete factors, for instance the extensively studied small molecules NO or endothelin-1, as well as numerous larger proteins, to modulate the normal cardiac function, pathophysiology of cardiac remodeling and HF development 16. This suggests that their physiological and thereby therapeutic importance may be greater than previously considered. Our recent study demonstrated that endothelial transcription factor, forkhead box P1 (Foxp1), regulated maladaptive cardiac remodeling through inhibition of TGFβ signals and controlled the progression of cardiac dysfunction 17.

Given the high prevalence of hypertension, and that hypertension is the biggest single contributor to the global burden of cardiovascular diseases, and longstanding hypertension can ultimately leads to HF 9, 18, thus TAC pressure overload model was used in this study to mimic the effects of hypertension and to characterize the impact of genetic or pharmacologic interventions on the progression of LV remodeling and LV function deterioration.

Simvastatin was reported to be a strong inducer of endothelial Kruppel-like factor 2 (Klf2), which is an important transcription factor in ECs helping to maintain the vascular homeostasis 19. Therefore, we examined whether EC-Klf2 mediates the protective effect of simvastatin on TAC-induced cardiac remodeling and dysfunction, and the detailed mechanism. Our previous study showed that simvastatin alleviated atherosclerotic plaque formation through regulation of EC-Klf2-Foxp1 mediated inhibition of chronic vascular inflammation and the Nlrp3 inflammasome activation 20. In this study, we explored whether the induction of EC-Klf2-Foxp1 transcription factor network mediates the protective effects of simvastatin on HF progression through the abrogation of the maladaptive cardiac remodeling in a murine TAC-induced HF model.

The study demonstrated that simvastatin attenuated the TAC-induced cardiac fibrosis and hypertrophy contributing to the improvement of cardiac dysfunction; however, in mice with Klf2 targeted delivery to the endothelial cells with RGD magnetic nanoparticle for EC specific Klf2 inhibition, or in EC-Foxp1 deletion mice, the protective effect of simvastatin was significantly reduced. Further study demonstrated that direct repression of EC-TGFβ1 by simvastatin, through induction of EC-Klf2 or through EC-Klf2 activation of EC-Foxp1, was involved in the reduction of the pathological cardiac fibrosis and hypertrophy in TAC pressure overload induced cardiac dysfunction. Our study confirmed the novel protective effects of simvastatin on TAC-induced pathological cardiac remodeling through regulation of Klf2-TGFβ1 or KLF2-Foxp1-TGFβ1 signal pathway in cardiac microvasculature ECs in addition to the previous reports of simvastatin's pleiotropic effects, which might provide an opportunity for simvastatin as a future prevention for HF.

## Methods

### Animal procedures

The male mice of C57BL/6J brought from shanghai SLAC laboratory Animal Co., Ltd, Foxp1^fl/fl^; Cdh5-Cre^ERT2^ (Foxp1^ECKO^) and Foxp1^wt/wt^; Cdh5-Cre^ERT2^ wild-type 17 littermate control of 10-14 weeks age were used. Tamoxifen (100 mg/kg body weight) was administrated by intraperitoneal injection every other day for a total four times to induce EC-specific Foxp1 deletion in Foxp1^ECKO^ mice prior to the experiments. All animal procedures were performed in accordance with the Institutional Animal Care and Use of Laboratory Animals approved by the Tongji University Institutional Animal Care and Use Committee. Mice were sacrificed by an overdose of anesthesia with pentobarbital sodium (100 mg/kg) intraperitoneally at the indicated time point.

### Transverse aortic constriction induced pathological cardiac remodeling mouse model

**Figure 1:**
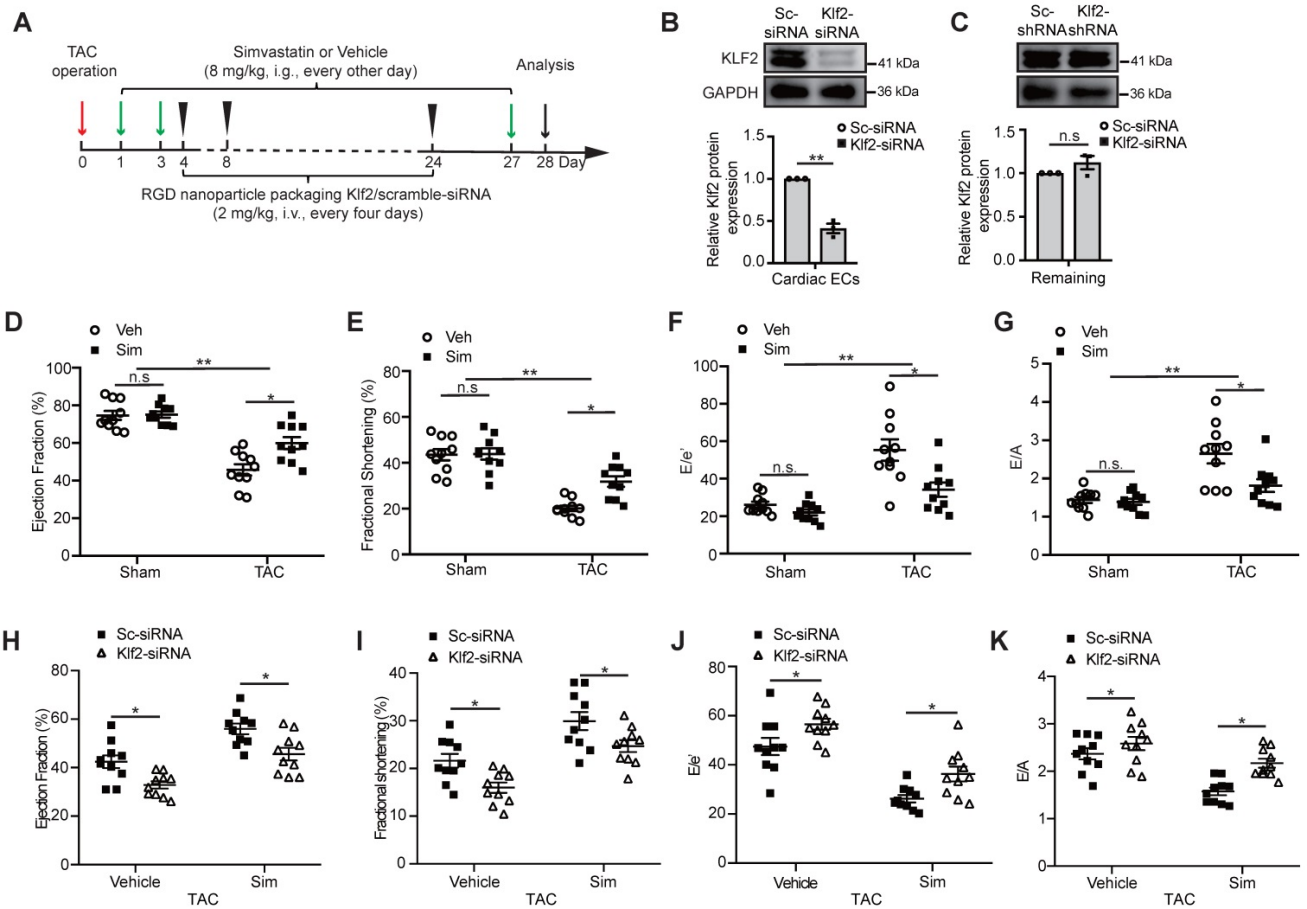
** Loss of simvastatin-mediated improvement of TAC-induced cardiac dysfunction after *in vivo* endothelial specific Klf2 inhibition. A,** A schematic figure of simvastatin administration and RGD-peptide magnetic nanoparticles endothelial target delivery of Klf2-siRNA in TAC left ventricle pressure overload mouse model. **B-C,** Western blot analysis shows that endothelial target delivery of Klf2-siRNA with RGD-peptide magnetic nanoparticles specifically inhibits the expression of Klf2 in cardiac ECs (B), but not in the other components of heart (C) (n=3). **D-G,** Echocardiogram analysis shows that simvastatin (Sim) elevates the TAC-induced reduction of left ventricle ejection fraction (LVEF) (D) and fractional shortening (LVFS) (E), and reduces the elevated ratio of E/e' (F) and E/A (G) compared with the vehicle (Veh) control (n=10). **H-K,** RGD-nanoparticle inhibition of EC-Klf2 expression *in vivo* reduces the simvastatin's improvement of the cardiac systolic dysfunction shown by LVEF (H) and LVFS (I), as well as diastolic function shown by E/e' ratio (J) and E/A ratio (K) (n=10). Data are means ± S.E.M.*P<0.05, ** P<0.01 and n.s not significant. Unpaired Student's t-test (B, C) and 2-way ANOVA with Turkey test (D-K).

TAC LV pressure overload model was performed as previously described 21. In brief, mice were anesthetized with isoflurane (2% vol/vol) with a rodent respirator device. The transverse aorta between the right innominate and left common carotid arteries were ligated against a blunted 27-gauge needle with a 7-0 suture. The needle was then gently removed. The sham procedure was identical except that the aorta was not ligated. Echocardiography was performed at 28 days after surgery and the data prior to surgery used as control. Simvastatin (8 mg/kg) or vehicle control 22 was administrated intragastrically every other day after TAC operation for 28 days (Figure 1A).

### Echocardiography Analysis

Echocardiography was performed to evaluate the cardiac geometry, and systolic and diastolic function as previously described 23. The Visual Sonics high-resolution Vevo2100 ultrasound system (VisualSonics Inc., Canada) with a 30-MHz linear array ultrasound transducer (MS-400, VisualSonics Inc.) was used. In brief, mice were anesthetized with isoflurane (2% vol/vol) with a rodent respirator device until the heart rate stabilized at 400 to 500 beats per min. Parasternal long-axis images were acquired in B-mode with appropriate position of the scan head to identify the maximum LV length. In this view, the M-mode cursor was positioned perpendicular to the maximum LV dimension in end-diastole and systole, and M-mode images were obtained for measuring wall thickness and chamber dimensions. LV ejection fraction (LVEF) and LV fractional shortening (LVFS) were calculated automatically. The apical four-chamber view was acquired and the peak flow velocities during early diastole (E wave) and late diastole (A wave) across the mitral valve, as well as early-diastolic peak velocity (e') of the mitral valve ring were measured. E/e' and E/A ratios, which reflected the left ventricular diastolic function, were calculated.

### Histology

After anesthetized with pentobarbital sodium (100 mg/kg, i.p.), the mice were perfused with cold PBS and hearts harvested, fixed with 4% paraformaldehyde (PFA) for 24 h, and embedded in paraffin wax or OCT. Serial sections were obtained at 6 µm intervals for paraffin embedded tissue and 8 µm intervals for OCT embedded tissue. The sections were stained with Masson's trichrome for detection of cardiac fibrosis and Alexa Fluor^TM^ 488 conjugated wheat germ agglutinin (WGA) (W11261, Invitrogen) for measurement of cardiomyocyte size *in vivo* 24. Images were captured by a Leica microscope (DM6000B, Leica, Germany). To quantify cardiac fibrosis, 10 fields were randomly selected from 3 cardiac sections and calculated as the percentage of Masson's trichrome positive-stained area to total myocardial area. Similar methods were used to evaluate cardiac hypertrophy by calculation of the average cardiomyocyte area in WGA staining heart sections.

Immunostaining was performed using the following antibodies: Mouse anti-α-smooth muscle actin (α-SMA) (5 µg/mL, A5228, Sigma-Aldrich), Rabbit anti-vimentin (5 µg/mL, #5741, Cell Signaling Technology), Mouse anti-PCNA (5 µg/mL, #2586, Cell Signaling Technology). After washing with PBST, the cells were incubated with Alexa Fluor 488-conjugated donkey anti-mouse secondary antibodies (2 µg/mL, A21206, Invitrogen) and Alexa Fluor 568- conjugated donkey anti-rabbit secondary antibodies (2 µg/mL, A10042, Invitrogen) for 45 min. Slides were mounted with Vectashield mounting medium containing DAPI (Vector Laboratories, Burlingame, USA). Images were captured by a Leica fluorescent microscope (DM6000B, Leica, Germany). The quantification of histological and immunofluorescence studies was performed by an observer blind to the experimental groups.

### Collection of mouse cardiac endothelial cells and culture

Mouse cardiac ECs were isolated as previously described 25. In brief, mice were anesthetized with pentobarbital sodium (100 mg/kg, i.p.) and the heart was excised aseptically and transferred to ice-cold Dulbecco's modified Eagle's medium (DMEM). The tissues were minced finely, followed by digestion in 1 mg/mL warm collagenase I (C5849, Sigma), 5 units/mL Dispase (345235, Collaborative) and 60 units/mL DNase I (10104159001, Roche) at 37 °C for 45 min with gentle agitation. The digested tissue was triturated using a 20-ml syringe with an 18-gauge cannula. The single cell suspension was then passed through a 70-µm cell strainer and subjected to centrifugation. The cell pellet was resuspended in 0.1% bovine serum albumin/PBS and incubated with anti-mouse CD31 (553370, BD pharmingen) antibody conjugated Dynabeads^TM^ (11035, Invitrogen) for 20 min with rotation. The Dynabeads were prepared according to the manufacturer's instructions. After separation in a magnetic separator, the cells were lysed by TRIzol^TM^ reagent and gene expression was quantified by RT-qPCR.

Mouse cardiac microvascular endothelial cell line (mCMVECs) were a kind gift from Dr. Ma from Wuxi School of Medicine, Jiangnan University 26, and passages between 4-8 were used in our study. Mouse cardiac fibroblast cells (MCFs) (M6300-57, ScienCell) were cultured in DMEM medium supplemented with 10% fetal bovine serum (Gibco, USA), Mouse Macrophages (MMa-bm) (M1920-57, ScienCell) were cultured in Macrophage Medium (MaM, #1921, ScienCell) and neonatal mouse cardiac myocytes (MCMs) (M6200-57, ScienCell) were cultured in DMEM medium supplemented with 15% fetal bovine serum (Gibco, USA). The cells at 3-8 passages were plated in 6-well plates and used for experiments.

### RNA extraction and RT-qPCR

For quantitative PCR (qPCR), RNA from the heart tissue, cardiac ECs and cultured cell lines was extracted by TRIzol^TM^ and cDNA synthesized by SuperScript First Strand Synthesis System (Invitrogen, USA). RT-qPCR was performed using PowerUp^TM^ SYBRTM Green master mix (A25741, Applied Biosystems) on a QuantStudio^TM^6 Flex Real-time PCR system (Applied Biosystems, USA) following the manufacturer's protocol. GAPDH was used as an internal control. Primers used for quantitative real-time PCR were included in Table S1.

### Western Blot Analysis

Cell pellets were extracted in RIPA lysis buffer (P0013C, Beyotime, China) containing protease inhibitor cocktail (539131, Calbiochem, USA). Protein concentrations were determined by a BCA kit (23225, Pierce, USA). Equal amounts of cell extracts (30 μg total protein) were electrophoresed on sodium dodecyl sulfate-polyacrylamide gels, and then transferred onto polyvinylidene difluoride membranes (Millipore, USA). The membranes were blocked in 5% non-fat dried milk in TBS-T at room temperature for 2 h and incubated with indicated primary antibodies at 4 °C overnight. The primary antibodies used in the study were: anti-α-SMA (1 µg/mL, A5228, Sigma-Aldrich), anti-collagen I (1 µg/mL, 600-401-103, Rockland), anti-collagen III (1 µg/mL, NB600-594, Novus), anti-Klf2 (2 μg/mL, ab203591, Abcam), anti-GAPDH (1 µg/mL, 5174, Cell signaling technology). The membranes were washed three times with TBS-T for 10 min, and then incubated with IRDye-680LT Goat Anti-Mouse IgG (H+L) (20 ng/mL,925-68020, Li-Cor, USA) or IRDye-800CW Goat Anti-Rabbit IgG (H+L) (20 ng/mL, 925-32211, Li-Cor, USA) at room temperature for 1 h. After washing in TBS-T for another three times, the membranes were detected by the Odyssey infrared imaging system (Li-Cor, USA). Experiments were repeated three times and the target protein level was quantified by Image J and normalized to internal control.

### MTT assays

Index for cell proliferation was measured by MTT assays. Briefly, MTT (M5655, Sigma-Aldrich, USA) was added to the cultured cells with a final concentration of 0.5 mg/mL, which were cultured for an additional 4 h at 37 °C. The supernatant was then aspirated, and 150 μl dimethyl sulfoxide (DMSO) was added to the cells and the formazan crystals were dissolved by shaking thoroughly for 10 min. The absorbance of each sample was measured under an automatic absorbance reader (Bio-Rad, 168-1150, USA) using a testing wavelength of 490 nm and a reference wavelength of 630 nm.

### Cell migration assay

Scratch wound healing assay was used for measuring cell migration. An equal number of cells were plated in 12-well plates to achieve 90% confluence monolayer and cells were cultured with FBS-reduced DMEM medium. A vertical wound was created by scratching with a 200-µl pipette tip and then the culture medium was changed to EC conditioned medium from different treatment groups. Images of the wound were captured at designated times to assess wound closure rate.

### Cell immunostaining

Cells cultured on sterile glass cover slips in 24-well plates were fixed with 4% paraformaldehyde (PFA) in PBS, washed and permeabilized with 0.1% Triton X-100 PBS. The cells were blocked with 1% goat serum for 1 h and incubated with primary antibodies: rabbit anti-sarcomeric α-actinin (5 µg/mL, ab9465, Abcam) overnight. After washing, the cells were incubated with Alexa Fluor 488-conjugated donkey anti-mouse secondary antibodies (2 µg/mL, A21206, Invitrogen) for 45 min. Then, the cover slips were mounted with Vectashield mounting medium containing DAPI (Vector Laboratories, Burlingame, USA). The immunostaining images were captured under a Leica fluorescent microscope (DM6000B, Leica, Germany).

### RGD-peptide magnetic nanoparticle target delivery of Klf2-siRNA to endothelial cells

The RGD-peptide nanoparticles were synthesized as previously reported with modification 27. In brief, sodium citrate water solution (CA, 5 mg/mL) was added to the cubic magnetic nanoparticles (cMNPs) ethanol solution (0.5 mg/mL) under sonication (8% amplitude for 90 min). Then, the product of cMNPs-CA was washed three times with deionized water and collected from magnetic separation. To improve the water solubility the polymer of polyethyleneimine (PEI, Mw=1000) was introduced to the cMNPs. cMNPs-CA (5 mg) were dispersed into deionized water, then NHS (0.6 mmol) and EDC (0.5 mmol) were added to activate the carboxyl groups on surface of cMNPs-CA for reaction with amino groups of PEI (cMNPs-PEI). Then cMNPs-PEI was functionalized with integrin RGD peptide (RGDRGDRGDRGDPGCL, 98.7%, Shanghai GL Biochem Ltd.) for good biocompatibility through the EDC/NHS coupling approach (cMNPs-RGD). Finally, cy5.5 NHS ester (1 mg in 0.3 mL DMSO) were mixed into the cMNPs solution (18 mg in 4 mL PBS) under vigorous vibration in dark at room temperature for 24 h.

The EC targeted delivery of Klf2-siRNA with RGD-peptide magnetic nanoparticles was performed as previously reported with modification 28. In brief, synthetic mouse Klf2-siRNA (Table S2) or scramble-siRNA control (GenePharma, China) was formulated with RGD-peptide and Cy5.5 containing core Fe_3_O_4_ magnetic nanoparticles at the ratio of 10 μg siRNA:50 μg nanoparticles in total 100 µl normal saline at room temperature for 1 h, thus the siRNA was wrapped into the inner interspace of the nanoparticles. The RGD-peptide magnetic nanoparticles containing Klf2-siRNA (2 mg/kg) were administrated by tail vein injection with a magnet on top of the chest at 4, 8, 12, 16, 20, 24 days following TAC. The RGD-peptide can function as an integrin specificity ligand leading the nanoparticles to concentrate in ECs, which can be detected by Cy5.5 and Perlus staining, providing an easy detection under fluorescence microscopy.

### Construct

Mouse full-length open reading frames of Klf2 were subcloned into pCMVTag2 vector as described previously 29. ShRNA of mouse Klf2 were inserted into pLKD-CMV-G and PR-U6-shRNA vector. The sequences of shRNA are listed in Table S3.

### Lentiviral package

Human embryonic kidney (HEK) 293T cells (ATCC, CRL-1573) were transfected with the vector plasmid, the lentiviral packaging constructs pCMV.DR8 and pMD2.G (Plasmid #12259, Addgene). The viral supernatants were harvested at 48 h and 72 h after transfection, filtered with 0.4-μm filters and applied to mouse cardiac microvasculature endothelial cell line (mCMVECs) in the presence of 8 µg/mL polybrene (Santa Cruz Biotechnology) for 6-8 h. The efficiency of infection was determined with fluorescence microscopy and successful Klf2 knockdown by Klf2-shRNA or overexpression by Lenti-Klf2 was determined by Western blot and RT-qPCR.

### Chromatin immunoprecipitation (ChIP) assays

The chromatin DNA was made from cardiac endothelial cells using a commercially available ChIP kit (Upstate Biotechnology, Inc.) and protein-G agarose beads were used to preclear chromatin. The isolated cardiac endothelial cells were cross-linked in 1% formaldehyde, and the previously characterized Klf2 polyclonal antibody or control rabbit IgG were used to immunoprecipitate protein-DNA complexes from chromatin. The reverse cross-linked chromatin was subjected to PCR using the primers listed in Table S4. The data presented are the average of five ChIP assays performed in triplicate.

### Luciferase Reporter Assay

The gene promoter sequences of TGFβ1, from -3515 to -2501 base pair containing 4 Klf2 binding sites were inserted into a pGL3 promoter luciferase vector. The primer sequences for amplification of Klf2 binding sequence are listed in Table S5 and the final sequence was validated by DNA sequencing. Luciferase activity was determined after transfection for 48 h using Dual-Luciferase assay kits (Promega, Madison, WI, USA). Individual luciferase activity was normalized to the corresponding renilla-luciferase activity, and the data presented are the average of five luciferase assays performed in triplicate.

### Reagents

Simvastatin (Selleck, S1792) was dissolved in DMSO, 4 mg/mL as a stock solution. For *in vivo* mice experiments a final dosage of 8 mg/kg was used (Figure 1A), and for *in vitro* cell culture experiments simvastatin was activated as previously described 30 and used at a final concentration of 1 μM in culture medium.

### Statistical Analysis

Data were presented as means ± S.E.M. for at least 5 independent assays unless otherwise noted. All data passed the normality and equal variance before analysis. Student's *t* test was used for 2-sample comparisons, 1-way ANOVA with Tukey post hoc tests for comparisons between multiple groups, and 2-way ANOVA for comparisons between multiple groups when there were 2 experimental factors. *P*< 0.05 was considered as statistically significant.

## Results

### Loss of simvastatin-mediated improvement of TAC pressure overload-induced cardiac dysfunction after *in vivo* endothelial specific Klf2 inhibition

Microvasculature ECs are the major non-myocyte population in total number in the adult mouse heart 15, and simvastatin was reported to be a strong inducer of Kruppel-like factor 2 (Klf2) in cultured ECs 19. We then tested the induction of Klf2 expression in cardiac ECs, fibroblasts, macrophages and cardiomyocytes following treatment of simvastatin and vehicle control, and the results confirmed that simvastatin induced the Klf2 expression mostly in ECs (Figure S1). Therefore, we further examined whether endothelial inhibition of Klf2 *in vivo* by targeted delivery of Klf2-siRNA with RGD-peptide magnetic nanoparticles into cardiac microvasculature ECs contributes to the improvement of simvastatin on TAC-induced cardiac dysfunction. The administration of the RGD-nanoparticles was described in Figure 1A and the efficiency and specificity of the RGD peptide-containing magnetic (Fe_3_O_4_) nanoparticle packaging Klf2-siRNA for specific blockade of genes in cardiac microvasculature ECs were described in our previous studies 17, 28. The results showed that RGD-nanoparticles were present mostly in cardiac microvascular ECs (Figure S2A), and significant inhibition of Klf2 expression in cardiac microvasculature ECs was confirmed in this study with no significant change of Klf2 expression in the remaining cardiac components when ECs were removed (Figure 1B-C and Figure S2B-C).

Echocardiography showed significant cardiac systolic dysfunction demonstrated by a reduction in LVEF (Figure 1D) and LVFS (Figure 1E), and cardiac diastolic dysfunction demonstrated by an increase in E/e' ratio (Figure 1F) and E/A ratio (Figure 1G) in the TAC hearts compared with the sham groups (Figure S3). Simvastatin significantly improved the TAC-induced cardiac systolic and diastolic dysfunction, while no significant change was observed between simvastatin and vehicle control in sham groups (Figure 1D-G). This significant improvement of cardiac dysfunction, including cardiac systolic function (Figure 1H-I) and the diastolic function (Figure 1J-K), was partially ablated after EC-specific inhibition of Klf2 expression* in vivo* with RGD-nanoparticles packaging Klf2-siRNA compared with the nanoparticles packaging control scramble-siRNA. These results suggest that EC-Klf2 might be important in mediating the protective effects of simvastatin on TAC-induced cardiac dysfunction.

### Lack of inhibitory effects of simvastatin on TAC-induced cardiac fibrosis and hypertrophy after *in vivo* endothelial specific Klf2 inhibition

**Figure 2:**
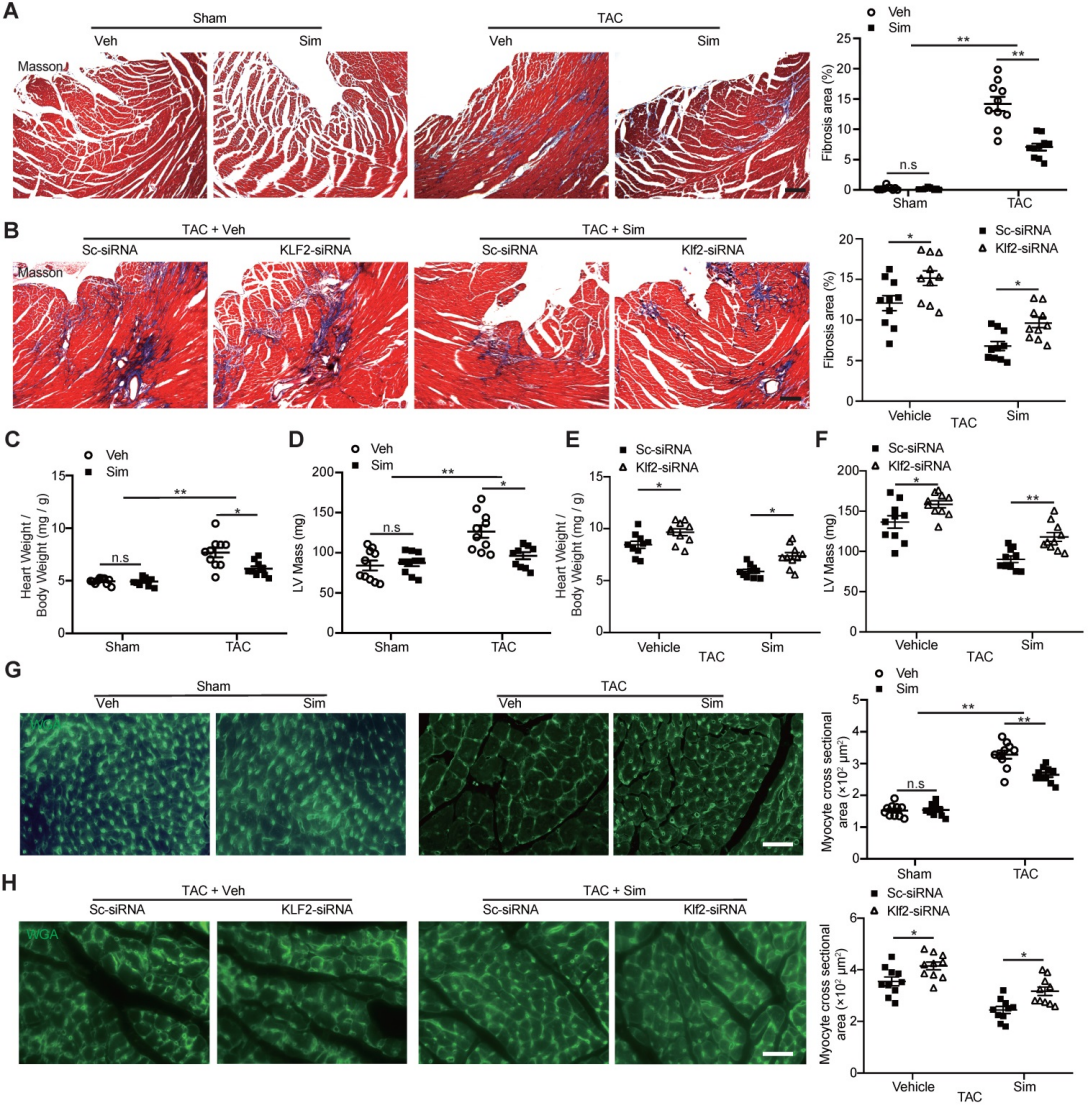
** Lack of inhibitory effects of simvastatin on TAC-induced cardiac fibrosis and hypertrophy after *in vivo* endothelial specific Klf2 inhibition. A-B,** Masson's Trichrome staining shows a significant reduction of cardiac fibrosis in TAC pressure overload mice following administration of simvastatin compared with the vehicle control (A), while RGD-nanoparticle* in vivo* inhibition of EC-Klf2 expression attenuates the simvastatin-mediated reduction of TAC-induced increased cardiac fibrotic area compared with the nanoparticles packaging control scramble (Sc)-siRNA (B), with representative image (left) and quantification (right) (n=10). **C-F,** Simvastatin reduces the TAC-induced elevation of heart to body weight ratio (C) and LV mass by echocardiogram (D) as compared with the vehicle control, while RGD-nanoparticle packaging Klf2-siRNA attenuated the TAC-induced elevated heart to body weight ratio (E), LV mass (F) compared with the nanoparticles packaging control scramble-siRNA (n=10). **G-H,** Wheat germ agglutinin (WGA) staining shows a significant decreased size of cardiomyocytes in TAC pressure overload mice following simvastatin administration compared with the vehicle control (G), while RGD-nanoparticle Klf2-siRNA attenuates the simvastatin-mediated reduction of TAC-induced increased cardiomyocyte size (H) with representative image (left) and quantification (right) (n=10). Data are means ± S.E.M. *P<0.05, ** P<0.01 and n.s. not significant. 2-way ANOVA with Turkey test. Scale Bars: A, B, 100 µm; G, H, 50 µm.

The TAC LV pressure overload is a well-studied mouse model of cardiac dysfunction characterized by the interstitial fibrosis and chamber remodeling 31. A significant increase of cardiac fibrotic area in TAC hearts was confirmed by Masson's trichrome staining compared with the sham operation, and simvastatin significantly inhibited the TAC-induced cardiac fibrosis compared with the vehicle control (Figure 2A). The significant inhibition of cardiac fibrotic area by simvastatin was attenuated after *in vivo* inhibition of Klf2 expression specifically in cardiac microvasculature ECs by RGD-nanoparticles packaging Klf2-siRNA compared with the nanoparticles packaging control scramble-siRNA (Figure 2B). Similarly, simvastatin significantly inhibited the TAC-induced elevation of heart-to-body weight ratio and LV mass (Figure 2C, D) whereas these effects of simvastatin were partially diminished by* in vivo* inhibition of EC-Klf2*.* (Figure 2E, F). Moreover, simvastatin significantly inhibited the TAC-induced increase of cardiomyocyte size (Figure 2G), which was also reversed by the specific inhibition of EC-Klf2 (Figure 2H). These results suggest that EC-Klf2 might contribute to the inhibitory effects of simvastatin on TAC-induced maladaptive cardiac remodeling.

### Endothelial Klf2 mediates the inhibitory effects of simvastatin on cardiac myofibroblast formation and fibroblast proliferation

**Figure 3:**
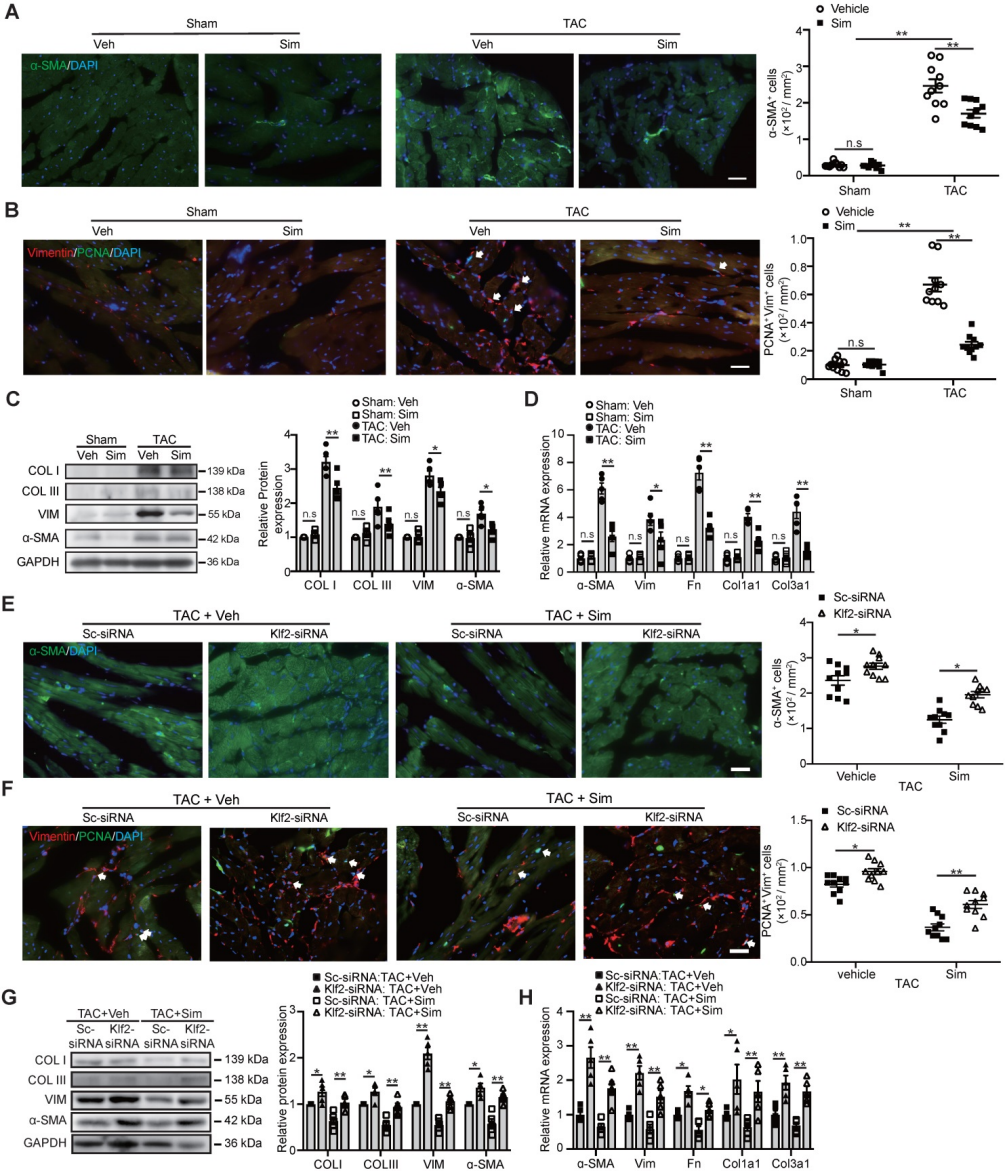
** Endothelial KLF2 mediates the inhibitory effects of simvastatin on TAC-induced cardiac myofibroblast formation and fibroblast proliferation. A-D,** Simvastatin decreases ɑ-SMA positive myofibroblast formation (A), reduces cardiac fibroblast proliferation shown by proliferating cell nuclear antigen (PCNA)/Vimentin (Vim) coimmunostaining (B) in TAC heart compared with the vehicle control, with no significant change in the sham mice (n=10). Simvastatin also reduced the and expression of α-SMA and collagen I (COL I), III (COL III) by western blot (C) and other extracellular matrix genes including vimentin (Vim), fibronectin (Fn), collagen type I alpha 1 subunit (Col1a1), collagen type III alpha 1 subunit (Col3a1) by RT-qPCR (n=5). (D) **E-H,** RGD-nanoparticle* in vivo* inhibition of EC-Klf2 expression reverses the simvastatin-mediated reduced α-SMA positive myofibroblasts (E), fibroblast proliferation (F), and the reduced expression of fibrotic genes, collagen type I, III by western blot (G) as well as other extracellular matrix genes by RT-qPCR (H) (n=5-10). Data are means ± S.E.M. *P<0.05, **P<0.01 and n.s not significant. 2-way ANOVA with Turkey test. Scale bars: A, B, E, F, 50 µm.

Pathological cardiac fibrosis is characterized by the formation of myofibroblasts and the acquisition of the myofibroblast phenotype endows the fibrotic cells with several capabilities including enhanced proliferation, migration, and excessive production and deposition of collagen and other extracellular matrix (ECM) proteins which reduce the cardiac compliance and accelerate the progression to cardiac dysfunction and eventually HF 6, 7. In this study we confirmed that simvastatin significantly reduced α-SMA positive myofibroblasts (Figure 3A) and inhibited fibroblast proliferation (Figure 3B) in the cardiac sections, as well as decreased the expression of α-SMA and type I and III fibrillar collagen as shown by western blot (Figure 3C) and other ECM proteins, including fibronectin, vimentin, and etc., as shown by RT-qPCR (Figure 3D) compared with the vehicle control. Lack of inhibitory effects of simvastatin on TAC-induced myofibroblast formation, enhanced fibroblast proliferation in the cardiac section, and the expression of α-SMA and collagen type I and III, as well as other ECM proteins (Figure 3E-H), were observed after* in vivo* specific inhibition of EC-Klf2 expression by target delivery of RGD-nanoparticles packaging Klf2-siRNA into cardiac microvasculature ECs.

**Figure 4:**
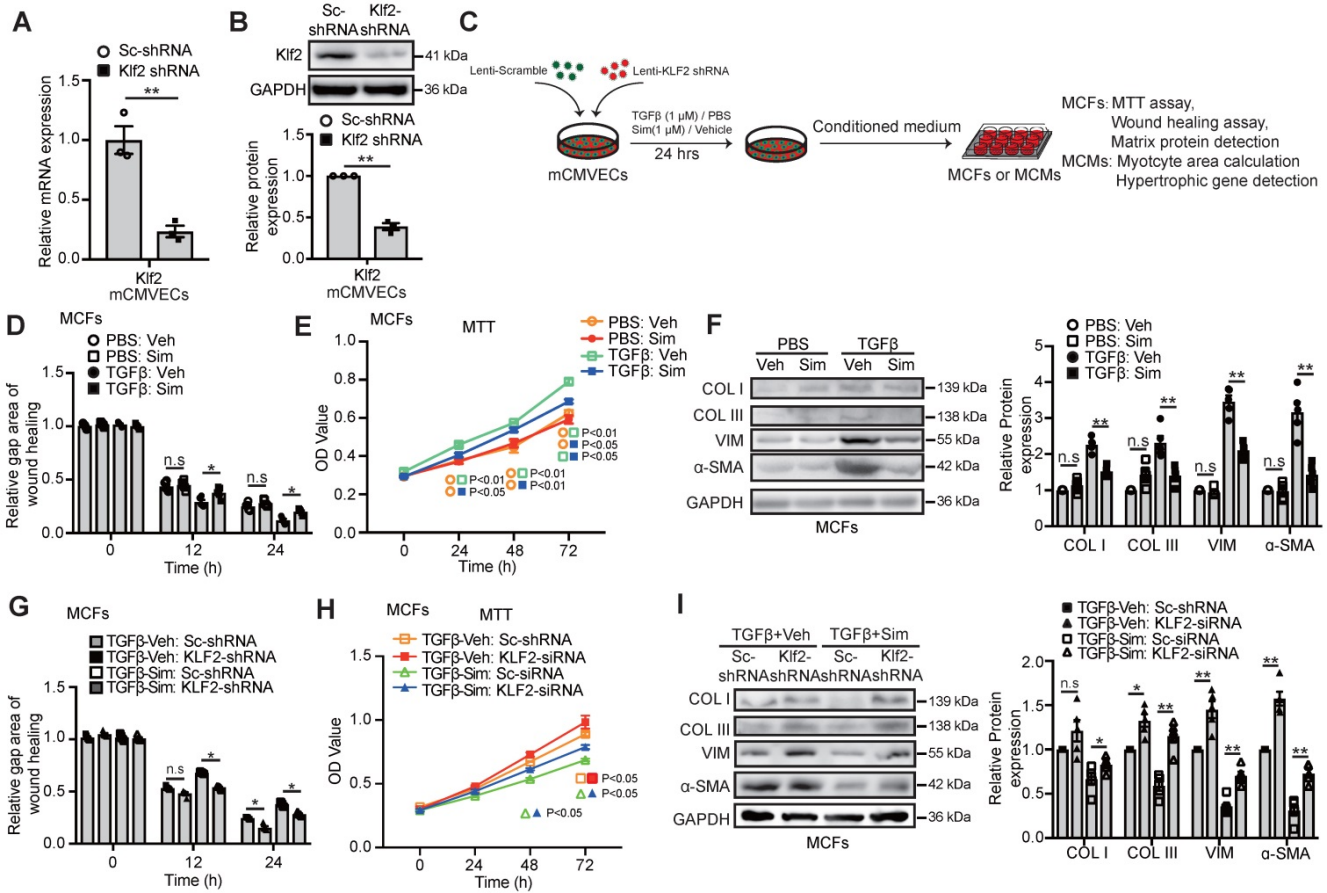
** Endothelial KLF2 mediates the inhibitory effects of simvastatin on TGFβ stimulated cardiac microvasculature endothelial cells condition medium (mCMVEC-CM) enhanced migration, proliferation and fibrotic gene expression of cardiac fibroblasts. A-B,** Klf2-shRNA significantly reduces the Klf2 expression in mCMVECs by RT-qPCR (A) and western blot (B) compared with scramble (sc)-shRNA treatment (n=3). **C,** Schematic diagram of an *in vitro* experiment to investigate the interaction between mouse cardiac microvascular endothelial cell line (mCMVECs) and mouse cardiac fibroblast (MCFs) or mouse cardiomyocytes (MCMs). **D-F,** The simvastatin treated mCMVEC-CM significantly inhibits migration of cardiac fibroblasts by wound healing assay (D), proliferation by MTT assay (E), and reduces their expression of α-SMA, and collagen I, III by western blot (F) compared with the vehicle treated mCMVEC-CM upon TGFβ stimulation, while no significant changes is observed in mCMVECs between simvastatin and vehicle treatment upon PBS stimulation (n=5). **G-I,** CM from Klf2-shRNA knockdown mCMVECs attenuates the simvastatin-mediated inhibition of increased migration of cardiac fibroblasts by wound healing assay (G), proliferation by MTT assay (H) and the simvastatin-mediated reduced expression of α-SMA, collagen type I, III by western blot (I) (n=5). Data are means ± S.E.M. *P<0.05, **P<0.01 and n.s not significant. Unpaired Student's t-test (A, B) and 2-way ANOVA with Turkey test (D-I).

In parallel with these *in vivo* findings, we used lentiviral Klf2-shRNA stable infected mouse cardiac microvascular endothelial cell line (Klf2-shRNA-mCMVECs) to determine whether inhibition of the Klf2 expression *in vitro* cultured ECs would result in the loss of simvastatin's inhibitory effects on cardiac fibroblast proliferation, migration and excessive ECM production. The successful Klf2 knockdown in Klf2-shRNA-mCMVECs was confirmed by RT-qPCR (Figure 4A) and western blot (Figure 4B).

We collected the conditioned medium (CM) from mCMVECs after simvastatin treatment with or without Klf2 knockdown (Figure 4C). As expected, mCMVEC-CM after simvastatin treatment significantly attenuated the enhanced migration (Figure 4D, Figure S4) and index of cell proliferation (Figure 4E) of TGFβ-treated mouse cardiac fibroblasts, with its parallel attenuation of the elevation of α-SMA positive myofibroblasts and the expression of α-SMA, collagen type I and III (Figure 4F) and other ECM proteins (Figure S5). Klf2 knockdown in mCMVECs significantly reduced the inhibitory effects of simvastatin on elevated fibroblast migration (Figure 4G, Figure S6) and index of cell proliferation (Figure 4H), associated with reduced expression of α-SMA, collagen type I, III **(**Figure 4I) and other ECM proteins (Figure S7) in the cultured cardiac fibroblasts.

To further elucidate that Klf2 mediates the effects of simvastatin on TGFβ signaling in ECs, mCMVECs were simulated with Angiotensin II, a strong inducer of TGFβ in the ECs 32 and involved in the TAC-mediated pathological cardiac remodeling. The results showed that AngII reduced Klf2 expression, but increased TGF-β1 expression in culture ECs and simvastatin reversed the AngII-mediated reduction of Klf2, but reduced the AngII-mediated elevation of TGF-β1 expression. The Klf2 inhibition by shRNA reversed the simvastatin-mediated reduction of AngII elevated TGF-β1 expression (Figure S8A), and Klf2 overexpression reduced AngII elevated TGF-β1 expression as simvastatin (Figure S8B-D). As expected, the conditioned medium from KLF2 overexpress mCMVECs attenuated the TGFβ induced cardiac fibroblast migration and proliferation with associated reduced the expression of α-SMA, vimentin, collagen type I, collagen type III (Figure S8E-G).

These results suggest that EC-Klf2 plays an important role in the inhibitory effects of simvastatin on cardiac fibroblast proliferation, migration and myofibroblast formation, as well as excessive production and deposition of ECM proteins in the heart, which contributes to the protective effects of simvastatin on TAC-induced pathological cardiac fibrosis.

### Endothelial KLF2 mediates the simvastatin's inhibitory effect on myocardial hypertrophy and cardiac hypertrophic gene expression

**Figure 5:**
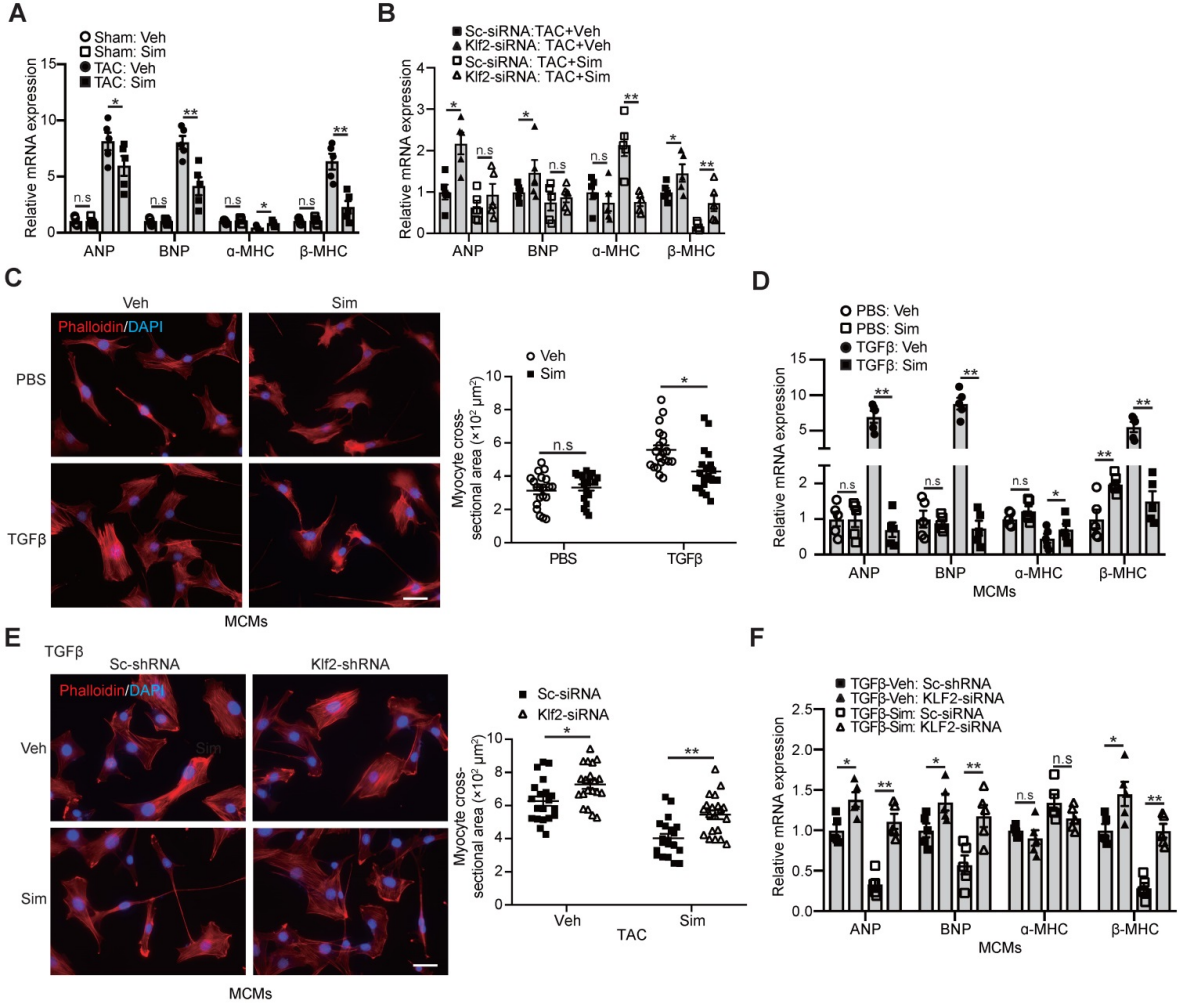
** Endothelial KLF2 mediates the inhibitory effects of simvastatin on increased size and fetal gene activation of cardiomyocytes. A-B,** Simvastain reduces the TAC-induced expression of cardiac fetal genes, such as ANP, BNP and immature cardiac gene β-MHC, and increases the expression of mature cardiac gene α-MHC by RT-qPCR (n=5) (A), whereas RGD-nanoparticle packaging Klf2-siRNA attenuates the simvastatin-mediated reduction of elevated expression of cardiac fetal genes, reduced α-MHC expression compared with the nanoparticles packaging control scramble-siRNA (n=5). (B). **C-D,** Simvastatin treated mCMVEC-CM significantly decreases the increased size of mouse cardiomyocytes by Phalloidin staining (C), with quantification on the right (20 cells/each group), and reduces their expression of hypertrophic genes by RT-qPCR (D) compared with vehicle treated mCMVEC-CM upon TGFβ stimulation (n=5). **E-F,** CM from lentiviral Klf2-shRNA knockdown mCMVECs attenuates the simvastatin-mediated reduced cardiomyocyte size (E) and expression of cardiac fetal genes (F) upon that of TGFβ stimulation compared with the CM from scramble-shRNA infected mCMVECs (n=5). Data are means ± S.E.M. *P<0.05, **P<0.01 and n.s not significant. 2-way ANOVA with Turkey test. Scale bars: **C,** E, 50 µm.

An important molecular feature of pathological hypertrophy is the induction of the molecular stress hypertrophic gene program that is classically absent in physiological hypertrophy 10. Significant expression of hypertrophic genes, such as atrial natriuretic peptide (ANP), brain natriuretic peptide (BNP), and β-myosin heavy chain (β-MHC), but reduced expression of mature cardiac gene ɑ-myosin heavy chain (ɑ-MHC), was observed in the TAC-heart, and simvastatin significantly reduced this hypertrophic gene expression (Figure 5A). The inhibitory effects of simvastatin on hypertrophic gene expression were absent after *in vivo* inhibition of Klf2 expression in cardiac microvasculature ECs by the RGD-nanoparticles packaging Klf2-siRNA compared with the nanoparticles packing control scramble siRNA (Figure 5B).

In parallel with these *in vivo* findings, we found a similar significant reduction of increased cardiomyocyte size (Figure 5C) and elevated expression of cardiac hypertrophic genes, ANP, BNP, β-MHC, but decreased ɑ-MHC expression (Figure 5D) in cardiomyocytes cultured with mCMVEC-CM upon TGFβ stimulation and simvastatin treatment compared with the simvastatin-vehicle control. Klf2 knockdown in mCMVECs significantly reduced the inhibitory effects of simvastatin on the reduction of increased cardiomyocyte size and hypertrophic gene expression (Figure 5E and 5F). Furthermore, Klf2 overexpression in mCMVECs significantly reduced the TGFβ stimulated mCMVECs-CM induced an increase in the expression of hypertrophic gene (Figure S8H).

These results suggest that EC-Klf2 plays an important role in the simvastatin's inhibitory effect on the cardiomyocyte size increase and hypertrophic gene expression, which contributes to the protective effects of simvastatin on TAC-induced pathological cardiac hypertrophy.

### Repression of TGFβ1 by Klf2 or Klf2-Foxp1 transcription factor network in endothelial cells mediates the inhibitory effects of simvastatin on TAC-induced maladpative cardiac remodeling

**Figure 6:**
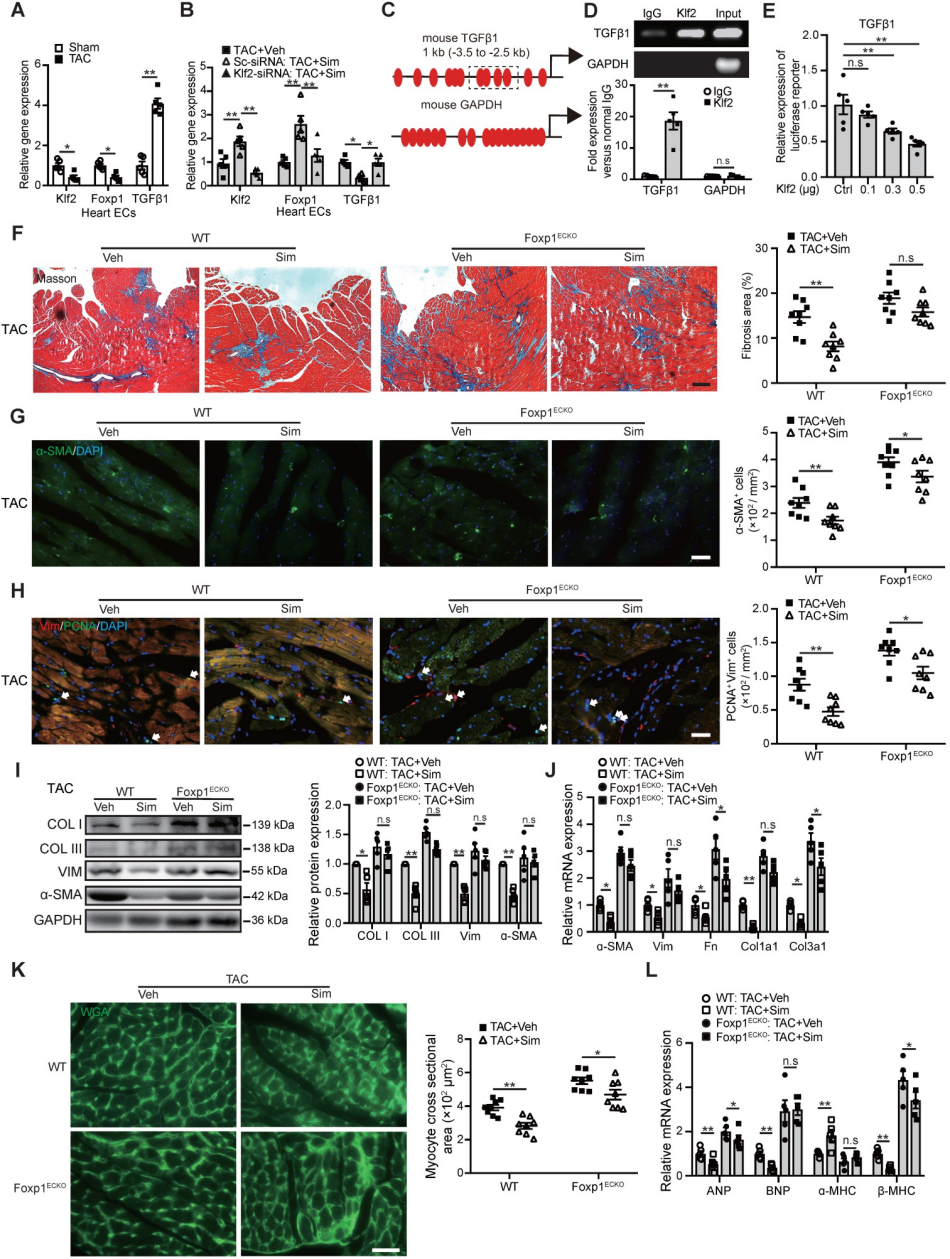
** Endothelial Klf2 or Klf2-Foxp1 transcription factor network directly represses TGFβ1 repression and mediates the inhibitory effects of simvastatin on TAC-induced pathological cardiac remodeling. A,** RT-qPCR shows a significant reduction of Klf2 and fork head box protein 1 (Foxp1) but increase of TGFβ1 expression in cardiac microvasculature ECs of TAC heart compared with those of sham group. (n=5) **B,** Simvastatin significantly elevates EC-Klf2 and EC-Foxp1 but reduces EC-TGFβ1 expression compared with the vehicle control, and specific inhibition of Klf2 expression in cardiac microvasculature ECs* in vivo* by RGD-nanoparticles packaging Klf2-siRNA reverses the simvastatin-medicated elevation of EC-Klf2 and EC-Foxp1, as well as reduction of EC-TGFβ1 expression compared with the vehicle control. (n=5) **C,** Schematic diagram of Klf2 binding sites in the promoter of TGFβ1. The dashed box indicates the sequences amplified for luciferase reporter assay. **D,** Chromatin immunoprecipitation (ChIP) assay shows association of Klf2 and TGFβ1 by ChIP-qPCR (bottom) with agarose gel in the top (n=5). **E,** Luciferase reporter assay of TGFβ1 promoter region containing the Klf2 binding sites in NIH-3T3 cells (n=5). **F,** Masson's Trichrome staining shows reduction of simvastatin-mediated inhibition of cardiac fibrosis in Foxp1^ECKO^ mutants compared with the littermate wild-type (WT) control mice, with representative image (left) and quantification (right) (n=8). **G-J,** EC-Foxp1 deletion reversal of the simvastatin-mediated inhibition of cardiac fibrosis correlates with the reversal of reduced α-SMA positive myofibroblast (G) (n=8), reduced fibroblast proliferation by PCNA/Vimentin double immunostaining (H) (n=8), and reduced expression of fibrotic genes, collagen type I, III and α-SMA by western blot (I) (n=5), as well as other ECM gene expression by RT-qPCR (J) (n=5). **K-L,** Endothelial specific Foxp1 deletion mice attenuates the simvastatin-mediated reduction of TAC-induced increased cardiomyocyte size by WGA staining (K) (n=8) and elevated expression of cardiac hypertrophic genes, ANP, BNP, β-MHC, but reduced α-MHC expression compared with the littermate wild-type control mice (L) (n=5). Data are means ±S.E.M. *P<0.05, **P<0.01 and n.s. not significant. Unpaired Student's t-test (A, D), 1-way ANOVA with Turkey test (B, E), 2-way ANOVA with Turkey test (F-L). Scale Bars: F, 100 µm; G, H, K, 50 µm.

Klf2 is an important transcription factor highly expressed in ECs and a previous study showed that Klf2 suppresses TGFβ signaling in the endothelium through induction of Smad7 and inhibition of activator protein 1 (AP-1), which was important for the maintenance of vessel homeostasis 33. In this study we found that microvasculature ECs from the TAC heart exhibited a significant reduction of Klf2 but an increase of TGFβ1 expression (Figure 6A). Simvastatin significantly elevated EC-Klf2 but reduced EC-TGFβ1 expression (Figure 6B and Figure S9). Furthermore, the specific inhibition of Klf2 expression in cardiac microvasculature ECs* in vivo* by the RGD-nanoparticles packaging Klf2-siRNA reversed the simvastatin-mediated reduction of EC-TGFβ1 expression (Figure 6B).

These results indicate that the Klf2 suppression of TGFβ1 mediated by simvastatin in ECs might contribute to the attenuation of *in vivo* pathological cardiac fibrosis and hypertrophy, and further lead to the improvement of progression of cardiac dysfunction to HF.

Sequence analysis showed Klf2 binding sites in the promoter region of mouse TGFβ1 (Figure 6C). ChIP-qPCR showed an association of Klf2 with the promoter of TGFβ1 (Figure 6D) and luciferase assay confirmed that Klf2 expression vector dose-dependently repressed the promoter of TGFβ1 containing Klf2 binding sites in NIH-3T3 cells (Figure 6E). These results suggest that Klf2 can directly regulate TGFβ1 expression and the preventive effect of simvastatin on cardiac remodeling and dysfunction could involve induction of EC-Klf2 which then represses EC-TGFβ1 expression.

Our previous study reported that simvastatin alleviated the atherosclerotic lesion formation through upregulation of EC-Klf2 and its downstream target gene Foxp1 20, and the elevated Foxp1 expression in cardiac microvasculature ECs could repress TGFβ signaling and improve the pathological cardiac remodeling 17. In this study, reduced Foxp1 expression was observed in microvasculature ECs of TAC hearts compared those of sham group (Figure 6A), and simvastatin significantly elevated EC-Foxp1 expression. However, EC-specific inhibition of Klf2 expression by the RGD-nanoparticles packaging Klf2-siRNA reversed the simvastatin-mediated elevation of EC-Foxp1 expression (Figure 6B). These results suggested that EC-Foxp1 might also engage in the preventive effects of simvastatin on TAC-induced cardiac remodeling and dysfunction, thus Foxp1^ECKO^ mutant mice were performed to examine the effects.

**Figure 7:**
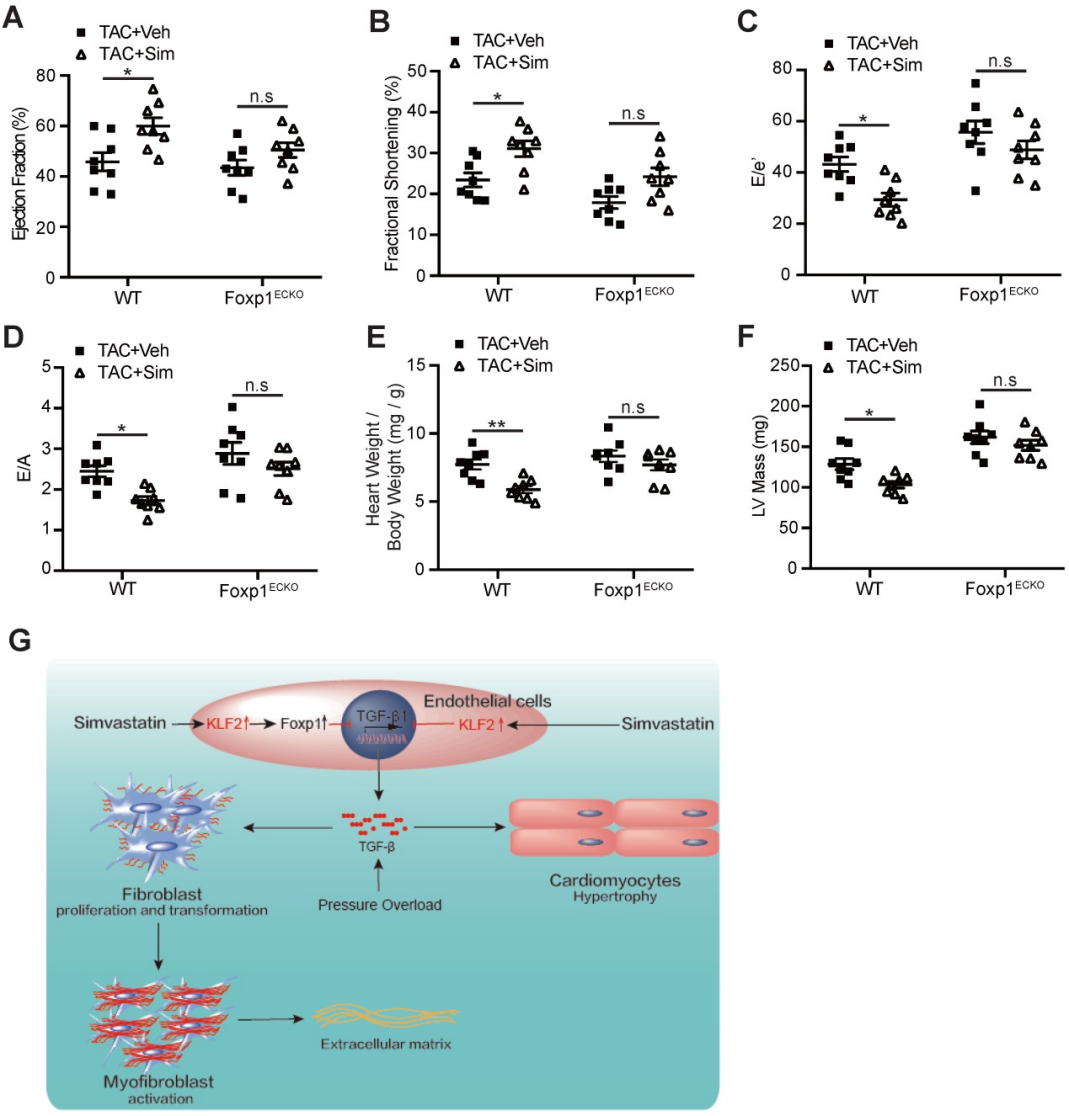
** Endothelial Foxp1 deletion attenuates the simvastatin-mediated improvement of the cardiac dysfunction. A-D,** Endothelial Foxp1 deletion mice exhibits reduction of the simvastatin improvement of the cardiac systolic dysfunction shown by LVEF (A) and LVFS (B), and the diastolic function shown by E/e' ratio (C) and E/A ratio (D) compared with the vehicle control (n=8). **E-F,** Endothelial Foxp1 deletion mice exhibits decrease of the TAC-induced elevated heart to body weight ratio (E), LV mass (F) compared with the vehicle control (n=8). **G,** The working model of the novel mechanism of simvastatin's prevention of TAC-induced cardiac remodeling through EC-KLF2 repression of EC-TGFβ1 or through EC-Klf2-Foxp1 transcription factor network, and therefore improves the progression of cardiac dysfunction to HF. Data are means ± S.E.M. *P<0.05, **P<0.01 and n.s. not significant. 2-way ANOVA with Turkey test (A-F).

The results indicated that the simvastatin-mediated inhibition of cardiac fibrosis was reduced in Foxp1^ECKO^ mutants compared with the littermate wild-type control mice (Figure 6F), with parallel reversal of the simvastatin-mediated inhibition of enhanced myofibroblast formation (Figure 6G), fibroblast proliferation (Figure 6H) and reduced expression of α-SMA, collagen type I and III (Figure 6I) and other ECM proteins (Figure 6J), as well as the reversal of simvastatin-mediated reduction of cardiomyocyte size (Figure 6K) and reactivation of cardiac fetal genes (Figure 6L). Furthermore, the simvastatin-mediated improvement of cardiac systolic and diastolic dysfunction was significantly attenuated in Foxp1^ECKO^ mutants compared to the littermate wild-type control mice (Figure 7A-F). Moreover, we found that no significant change of EC-Klf2 expression between vehicle control WT and EC-Foxp1 deletion mice but simvastatin increased EC-Klf2 expression in both WT and EC-Foxp1 deletion mice. However, simvastatin significantly reduced EC-TGF-β1 expression, with less but still significant reduction in EC-Foxp1 deletion mice (Figure S10). Taken together, we conclude that simvastatin prevents TAC-induced pathological cardiac remodeling via direct repression of TGFβ1 through induction of EC-Klf2 or by Klf2-Foxp1 transcription factor network to further retard the progression of cardiac dysfunction to HF, which was shown in the schematic Figure 7G.

## Discussion

The major findings of the present study were that EC-Klf2 signal mediates the inhibitory effects of simvastatin on pathological myocardial fibrosis and hypertrophy and improvement of cardiac dysfunction via direct suppression of TGFβ1 or through EC-Klf2-Foxp1-TGFβ1 pathway, thus revealing a novel cholesterol lowering independent mechanism for simvastatin in the prevention of cardiac dysfunction progression to HF during the process of myocardial remodeling in the TAC pressure overloading model.

Cardiomyocytes are terminally differentiated cells and have the negligible regenerative capacity, thus the repair processes involve cardiomyocyte hypertrophy and fibrotic scar tissue replacement. Left ventricular hypertrophy is more commonly associated with hemodynamic overload imposed by pressure such as hypertension than with volume overload. However, the mechanism is not clear and the therapy is unsatisfactory. Statins were reported to effectively reverse the hypertrophic states of the myocardium and retard the remodeling process to HF through their pleiotropic effects independent of cholesterol-lowering, including inhibition of p21 ras activation 13 or a small GTP-binding protein, GDP dissociation stimulator, mediated inhibition of Rac1, Rho kinase, and extracellular signal-regulated kinase 1/2 pathways 14. In this study, we found that simvastatin induced Klf2 expression in endothelium which repressed TGFβ to reduce the size of cardiomyocytes and reactivate the fetal genes, finally contribute to the amelioration of LV hypertrophy in the TAC model.

Pathological cardiac fibrosis results from an unrestrained tissue repair process orchestrated predominantly by the myofibroblasts, the activated fibroblasts characterized by the appearance of α-SMA and over-production of ECM proteins. Statin treatment was reported to reduce the formation of geranylgeranyl pyrophosphate, which served as a lipid membrane anchor for proteins of Rho GTPase family, especially RhoA and decreases its downstream effector, Rho-associated kinase (ROCK) to limit the cardiac fibrosis in mouse ischemic reperfusion models through regulation of fibroblast proliferation and migration 12, 34. Recently, Marrone et al emphasized the link between Rho GTPases and the downstream effector Klf2 in hepatic stellate cells involving in liver fibrosis, thus providing new molecular insights into the mechanisms of underlying the beneficial effects of statins in liver fibrosis 35.

However, the benefit of statins is more controversial in HF patients. The MRC/HPS study treatment of HF patients with 40 mg simvastatin daily showed a non-significant trend toward lower HF deaths rate due to any cause and a marginally significant reduction in first hospital admission for worsening HF or HF death 36, 37. Analysis of endomyocardial biopsy material revealed statin-treated HF patients to have similar myocardial collagen deposition compared to non-statin-treated HF patients 38. These human studies of statins in HF involved patients with HF, whereas the current study focused on the preventive effects of simvastatin on HF development, the cardiac dysfunction during the cardiac remodeling process to HF.

TGFβ1, the key driving forces culminating in fibrosis, was reported initially to be derived from immune cells and subsequently produced by myofibroblasts and has been identified as a primary and potent mediator of myofibroblast transformation and fibrotic remodeling in the heart 7, 39. All cells presented in the myocardium can secrete autocrine and paracrine factors that modulate the function of neighboring cells.

Cardiac microvasculature ECs, the major non-myocyte population in the adult heart 15, secrete a wide variety of factors and play a crucial role in the regulation of cardiac fibrosis and hypertrophy 16, 40. Simvastatin, a strong inducer of Klf2 in the endothelium, has been reported to control endothelial identity and vascular integrity 41 by direct and indirect regulation of expression of over a thousand genes 42. In the current study, we discovered that simvastatin induced Klf2 expression which directly repressed the enhanced TGFβ1 expression in cardiac microvasculature ECs of TAC induced fibrotic heart to reduce fibroblast proliferation, migration and myofibroblast formation, as well as ECM production, thus ameliorated the pathological cardiac fibrosis, and further improved the cardiac dysfunction induced by the TAC operation.

We recently reported that simvastatin alleviates the atherosclerotic plaque formation via the upregulation of EC-Klf2 and its downstream target Foxp1 20, and this elevated Foxp1 expression in the endothelium inhibited the TGFβ signaling which regulated the pathological cardiac remodeling 17. In this study, we showed that genetic deletion of EC-Foxp1 limited the simvastatin-mediated protective effect on pathological cardiac fibrosis and hypertrophy, demonstrating that the EC-Klf2-Foxp1-TGFβ1 transcription network is necessary for the protective effects of simvastatin against TAC pressure overload-induced cardiac remodeling *in vivo*.

This study has several limitations including that Klf2 silence by RGD-nanoparticle did not fully block the effects of simvastatin, possibly due to other alternative pathways involved in the pleiotropic effects of simvastatin including the inhibition RhoA and ROCK activity on fibroblast proliferation, myofibroblast transformation and myocardium apoptosis, and Rac1 signal on NADPH oxidase and reactive oxygen species activity 12. Also, the male mice were exclusively performed in the *in vivo* experiments.

In conclusion, this study demonstrates that simvastatin inhibits TGFβ in the endothelium by direct Klf2-meidated repression or through the endothelial Klf2-Foxp1 transcription factor network, and thereby alleviates the cardiac fibrosis associated with fibroblast proliferation, myofibroblast formation and ECM protein production, and ameliorates the pathological cardiac hypertrophy with an increase of cardiomyocyte size and reactivation of cardiac fetal genes. The simvastatin-mediated improvement of pathological cardiac remodeling further attenuates the progression of cardiac dysfunction to HF. The clarification of the novel cardioprotective mechanism of simvastatin, the anti-remodeling effects of statin is mediated through EC-KLF2 direct repression of TGFβ1, or through the Klf2-Foxp1-TGFβ1 pathway, might provide an opportunity for simvastatin as a future intervention for prevention of HF.

## Supplementary Material

Supplementary figures.
