# Supplementary material for: Endothelial Klf2-Foxp1-TGFβ signal mediates the inhibitory effects of simvastatin on maladaptive cardiac remodeling
Source: Theranostics. 2021 Jan 1;11(4):1609–25. doi: 10.7150/thno.48153 (PMC7778601; doi:10.7150/thno.48153)

SUPPLEMENTARY MATERIAL

Endothelial Klf2-Foxp1-TGFβ signal mediates the inhibitory effects of simvastatin on maladaptive cardiac remodeling

Running Title: Li et al.; Endothelial Klf2-Foxp1 and simvastatin's cardiac remodeling prevention

Hongda Li, BSc[1,†]; Yanfang Wang, MD[1,†]; Jiwen Liu, MD[1,†]; Xiaoli Chen, MD[1]; Yunhao Duan, BSc[1]; Xiaoyu Wang, MD[1]; Yajing Shen, PhD[3]; Yashu Kuang, MD[1]; Tao Zhuang, PhD[1]; Brain Tomlinson, PhD[4]; Paul Chan, MD, PhD[5]; Zuoren Yu, MD, PhD[1]; Yu Cheng, PhD[3]; Lin Zhang, MD, PhD[1]; Zhongmin Liu, MD, PhD[1]; Yuzhen Zhang, MD, PhD[1]; Zhenlin Zhao, PhD[6,*]; Qi Zhang, MD, PhD[2,*]; Jie Liu, PhD[1,6,*]

[1]Key Laboratory of Arrhythmias of the Ministry of Education of China, Research Center for Translational Medicine, Shanghai East Hospital, Tongji University School of Medicine, Shanghai, China;

[2]Department of Cardiology, Shanghai East Hospital, Tongji University School of Medicine, Shanghai, China;

[3]Institute for Biomedical Engineering and Nano Science, Tongji University School of Medicine, Shanghai, China;

[4]Faculty of Medicine, Macau University of Science and Technology, Macau SAR, China;

[5]Division of Cardiology, Department of Internal Medicine, Wan Fang Hospital, Taipei Medical University, Taipei, Taiwan;

[6]Shenzhen Ruipuxun Academy for Stem Cell and Regenerative Medicine, Shenzhen, China.

† These authors contributed equally to this work.

*To whom correspondence should be addressed:

Jie Liu, PhD
Key Laboratory of Arrhythmias of the Ministry of Education of China
Research Center for Translational Medicine
Shanghai East Hospital, Tongji University School of Medicine,
150 Jimo Rd, Pudong New District, Shanghai, 200120, China
Tel:86-21-61569673
E-mail: kenliujie@126.com

Qi Zhang, MD, PhD
Department of Cardiology,
Shanghai East Hospital, Tongji University School of Medicine,
150 Jimo Rd, Pudong New District, Shanghai, 200120, China
E-mail: zhangqnh@hotmail.com

Zhenlin Zhao, PhD
Shenzhen Ruipuxun Academy for Stem Cell and Regenerative Medicine
14 Jinhui Rd, Pingshan New District, Shenzhen, 518122, China
E-mail: zhaozl2006@163.com

Supplementary Tables:

Supplementary Table 1: Primer sequence for real time quantitative PCR (RT-qPCR)

Gene Name	Primer Sequence	
α-SMA-mus	Forward	5’-GTCCCAGACATCAGGGAGTAA-3’
	Reverse	5'-TCGGATACTTCAGCGTCAGGA-3’
Vimentin-mus	Forward	5'-GGATCAGCTCACCAACGACA-3’
	Reverse	5’-AAGGTCAAGACGTGCCAGAG-3’
Fibronectin-mus	Forward	5’-TTCAAGTGTGATCCCCATGAAG-3’
	Reverse	5’-CAGGTCTACGGCAGTTGTCA-3’
Col1α1-mus	Forward	5’-GCTCCTCTTAGGGGCCACT-3’
	Reverse	5’-ATTGGGGACCCTTAGGCCAT-3'
Col3α1-mus	Forward	5’-CTGTAACATGGAAACTGGGGAAA-3'
	Reverse	5’-CCATAGCTGAACTGAAAACCACC-3'
ANP-mus	Forward	5’-GCTTCCAGGCCATATTGGAG-3’
	Reverse	5’-GGGGGCATGACCTCATCTT-3’
BNP-mus	Forward	5’-GAGGTCACTCCTATCCTCTGG-3’
	Reverse	5’-GCCATTTCCTCCGACTTTTCTC-3’
Myh6-mus	Forward	5’-GCCCAGTACCTCCGAAAGTC-3’
	Reverse	5’-ATCAGGCACGAAGCACTCC-3’
Myh7-mus	Forward	5’-TGGGGCAGAGCTTCAGTTG-3’
	Reverse	5’-TGGGGCAGGAGAAACTTTGC-3’
TGFβ1-mus	Forward	5’-AGCTGCGCTTGCAGAGATTA-3’
	Reverse	5’-AGCCCTGTATTCCGTCTCCT-3’
Klf2-mus	Forward	5’-CGCCTCGGGTTCATTTC-3’
	Reverse	5’-AGCCTATCTTGCCGTCCTTT-3’
GAPDH-mus	Forward	5’-AGGTCGGTGTGAACGGATTTG-3’
	Reverse	5’-GGGGTCGTTGATGGCAACA-3’

Supplementary Table 2: Sequence of gene-siRNA

Gene	Sequence
Scramble	5’-UUCUCCGAACGUGUCACGUTT-3’<br>5’-ACGUGACACGUUCGGAGAATT-3’
Klf2-mus	5’-GACCCUUUCAGUGCCACUUTT-3’<br>5’-AAGUGGCACUGAAAGGGUCTT-3’

Supplementary Table 3: Sequence for gene-shRNA

Gene	Sequence
Scramble	5’-TTCTCCGAACGTGTCACGT-3’
Klf2-mus	5’-GCACGGATGAGGACCTAAA-3’

Supplementary Table 4: Primer sequence for chromatin immunoprecipitation (ChIP) assays

Promoter	Primer Sequence	
TGFβ1	Forward	5’-CTGCAAAGGCACTAAGACT-3‘
	Reverse	5’-CAACGTATCACCCTCACAA-3‘
GAPDH	Forward	5’- CCCTACCCCACCTCCATTAT-3’
	Reverse	5’- TGGTGGGTGGAGTTTTCTTC-3’

Supplementary Table 5: Primer sequence for amplification of Klf2 binding promoter sequence in luciferase reporter assays

Gene	Primer Sequence	
TGFβ1	Forward	5’-AGGGTGATACGTTGGAAG-3‘
	Reverse	5’-GCCACTAGAAACCTAACGA-3‘

Supplementary Figure legends

Supplementary Figure 1. Mouse cardiac microvascular endothelial cells (mCMVECs), cardiomyocytes (MCMs), fibroblasts (MCFs) and macrophages (MMa-bm) were treated with simvastatin (1 μM) or vehicle control for 24 hrs. RT-qPCR shows simvastatin induced a robust elevation of Klf2 expression ECs, but only with mild elevation in macrophages and no significant changes of Klf2 expression in cardiomyocytes and fibroblasts (n=5). Data are means ± S.E.M. ***P*<0.01, * *P*<0.05 and n.s. not significant. Unpaired Student's t-test.

Supplementary Figure 2. Successful target delivery of KLF2-siRNA by RGD-peptide magnetic nanoparticles into cardiac microvascular endothelial cells for KLF2 inhibition *in vivo*. A, RGD-Fe3O4 magnetic nanoparticles are successfully delivered to cardiac microvascular ECs shown by Cy5.5 label (arrow head, 3rd panel) and Perlus staining of Fe (4th panel) containing in the nanoparticles in cardiac sections. **B-C,** RGD nanoparticle packaging KLF2-siRNA target delivery reduces the expression of KLF2 by RT-qPCR in cardiac ECs (B), with no significant change of KLF2 expression in the remaining cardiac components when ECs were removed (C) (n=5). Data are means ± S.E.M. ** P<0.01 and n.s. not significant. Unpaired Student's t-test. Scale bar: A: 20 μm.

Supplementary Figure 3. Representative images of echocardiography from the mouse groups. A-C, Representative images from the mouse groups of M Model across the Parasternal long axis, pulsed wave Doppler (PWD) across the mitral flow and tissue Doppler (TD) of mitral vavle ring at the 28th day after TAC or sham operation.

Supplementary Figure 4. The representative image of simvastatin treated mouse cardiac microvascular endothelial cell line (mCMVECs) conditioned medium (CM), mCMVEC-CM, significantly inhibits the TGFβ stimulated mCMVEC-CM enhanced cardiac fibroblast migration by wound healing assay, with no significant change between simvastatin and vehicle treatment upon PBS stimulation (n=5). Scale bars: 100 μm.

Supplementary Figure 5. The simvastatin treated mCMVEC-CM significantly reduces the expression of other extracellular matrix (ECM) genes such as vimentin (Vim) and fibronectin (Fn), collagen type I alpha 1 subunit (Col1a1), collagen type III alpha 1 subunit (Col3a1) compared with vehicle treated mCMVEC-CM upon TGFβ stimulation, while no significant changes were observed in mCMVECs between simvastatin and vehicle treatment upon PBS stimulation (n=5). Data are means ± S.E.M. ***P*<0.01 and n.s. not significant. 2-way ANOVA with Turkey test.

Supplementary Figure 6. The representative image of the condition medium (CM) from Krüppel like factor (Klf2) knockdown lentiviral Klf2-shRNA infected mCMVECs attenuates the simvastatin-mediated inhibition of increased cardiac fibroblast migration by wound healing assay upon TGFβ stimulation (n=5). Scale bars: 100 µm.

Supplementary Figure 7. CM from Klf2 knockdown lentiviral Klf2-shRNA infected mCMVECs attenuates the simvastatin-mediated reduced expression of extracellular matrix (ECM) genes such as vimentin (Vim) and fibronectin (Fn), collagen type I alpha 1 subunit (Col1a1), collagen type III alpha 1 subunit (Col3a1) in cardiac fibroblasts upon that of TGFβ stimulation compared with the CM from scramble-shRNA infected mCMVECs (n=5). Data are means ± S.E.M. *$P<0.05$ and **$P<0.01$. 2-way ANOVA with Turkey test.

Supplementary Figure 8. Klf2 mediates the inhibitory effect of simvastatin on AngII-induced TGFβ1 expression in mCMVECs and Klf2 overexpression inhibits TGFβ-induced mCMVEC-CM enhanced migration, proliferation and fibrotic gene expression of cardiac fibroblasts and attenuated the fetal genes reactivation in cardiomyocytes. A, RT-qPCR results showed that AngII reduces Klf2 expression, but increases TGFβ1 expression in mCMVECs and simvastatin reverses the AngII-mediated reduction of Klf2, but reduces the AngII-mediated elevation of TGF-β1 expression. And the Klf2 inhibition by shRNA reverses the simvastatin-mediated reduction of AngII elevated TGF-β1 expression (n=3). **B-C,** Klf2-overexpression lentivirus vector (Lenti-KLF2) significantly elevates the Klf2 expression in mCMVECs by RT-qPCR **(B)** and western blot **(C)** compared with scramble vector infection (n=3). **D,** RT-qPCR results showed significantly downregulated AngII-stimulated TGF-β1 expression in KLF2 overexpressing ECs. (n=3) **E-G,** Klf2 overexpression in mCMVECs attenuates the TGF-β1 stimulated mCMVECs-CM induced cardiac fibroblast proliferation by MTT assay **(E)**, migration by wound healing assay **(F)** and reduced the expression of α-SMA, vimentin (Vim), collagen type I (Cola1), colleagen type III (Cola3) by RT-qPCR **(G)** (n=3)**. H,** Klf2 overexpression in mCMVECs attenuates the TGF-β1 stimulated mCMVECs-CM induced elevation of cardiac fetal genes, such as ANP, BNP and immature cardiac gene β-MHC, and increases the expression of mature cardiac gene α-MHC

in cardiomyocytes by RT-qPCR (n=3). Data are means ± S.E.M. *P<0.05, **P<0.01 and n.s not significant. 2-way ANOVA with Turkey test (A) and unpaired Student's t-test (B-H).

Supplementary Figure 9. Simvastatin induces KLF2 protein expression in both sham and TAC heart ECs as shown by representative western blot figures in the top and quantification in the bottom (n=3). Data are means. Data are means ± S.E.M. *P<0.05 and **P<0.01 and 2-way ANOVA with Turkey test.

Supplementary Figure 10. RT-qPCR analysis of cardiac microvasculature ECs from simvastatin or vehicle treated Foxp1^{ECKO} mutants and wild-type (WT) littermates 28 days after TAC surgery. The results show EC-Foxp1 deletion exhibited no significant change of EC-Klf2 expression but blunted the simvastatin reduced EC-TGF-β1 expression compared with WT mice (n=3). Data are means ± S.E.M. *P<0.05 and **P<0.01 and n.s not significant and 2-way ANOVA with Turkey test.

Supplementary Figure 1

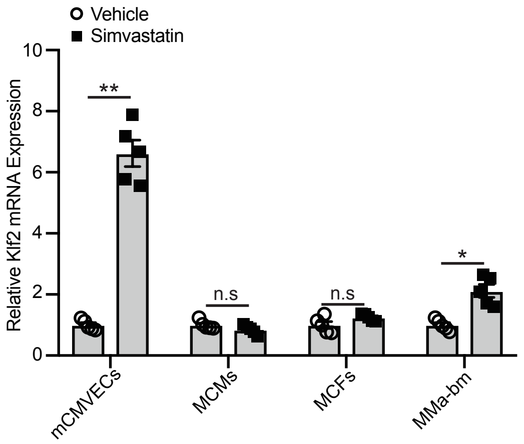

Supplementary Figure 2

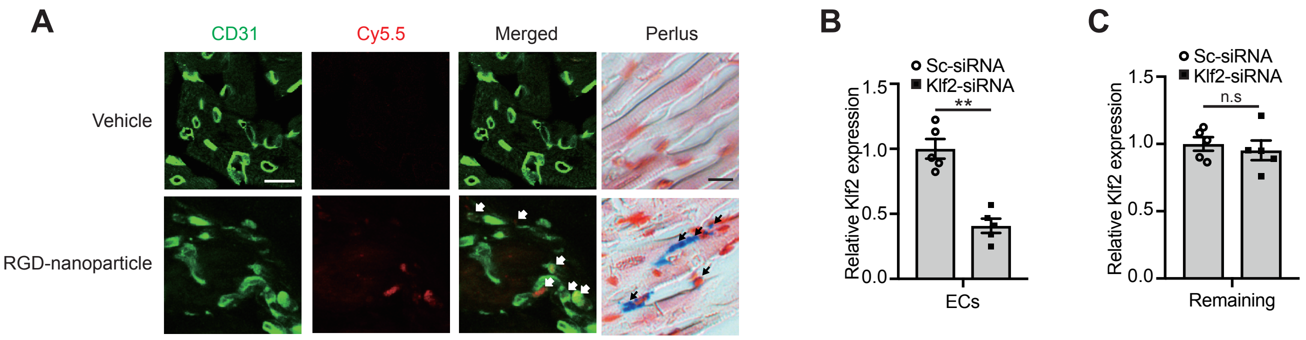

Supplementary Figure 3

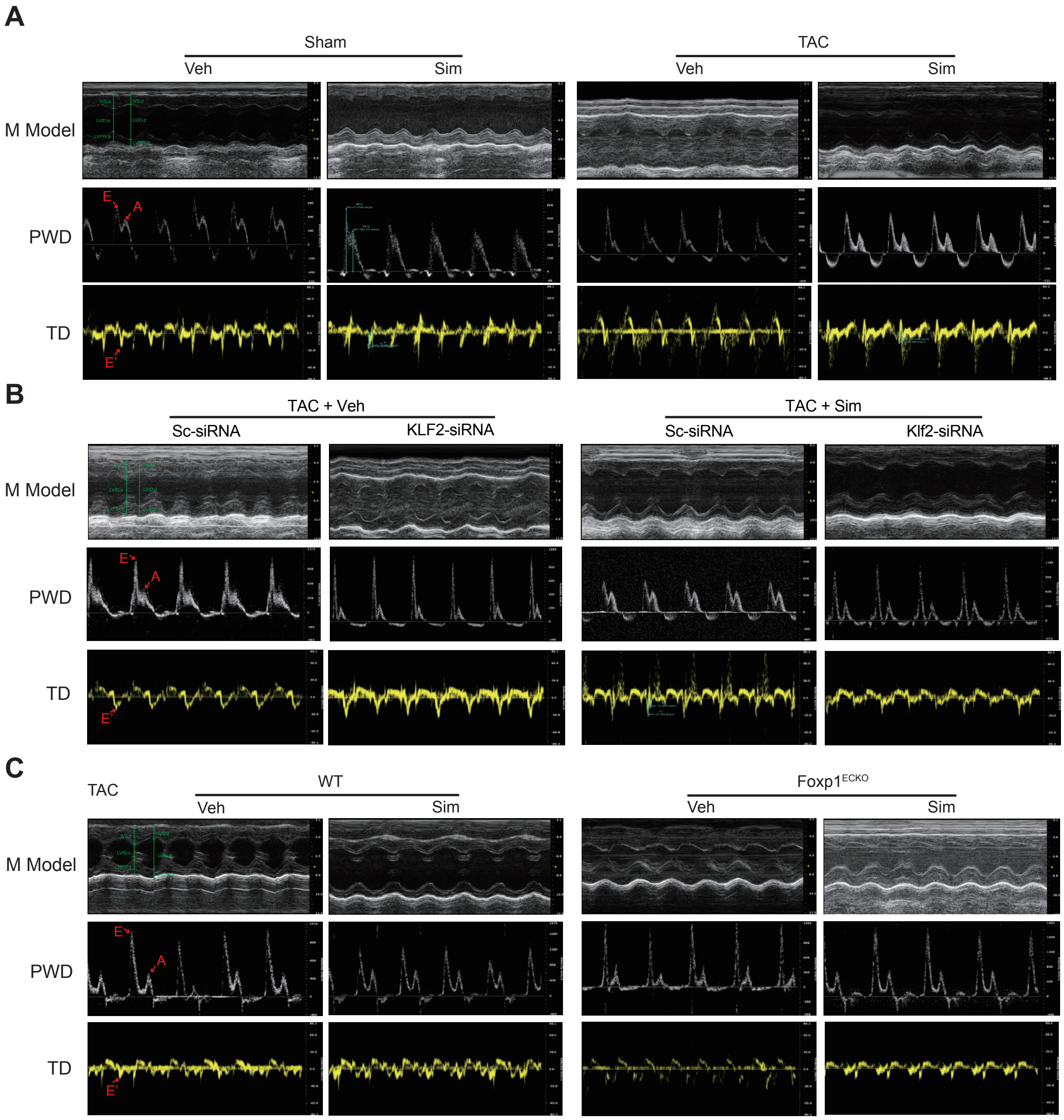

Supplementary Figure 4

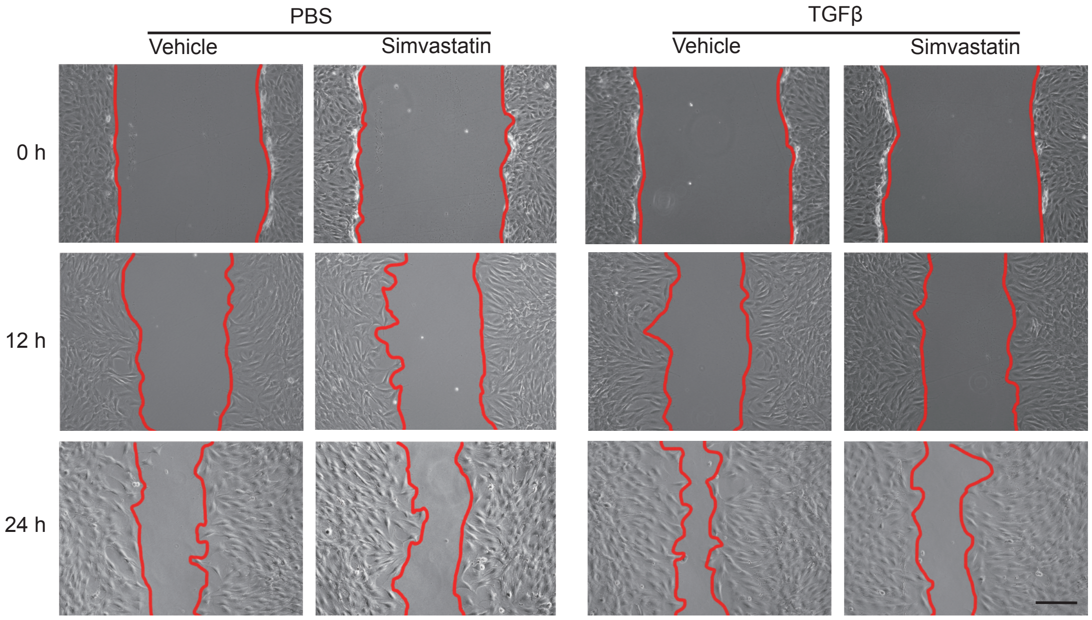

Supplementary Figure 5

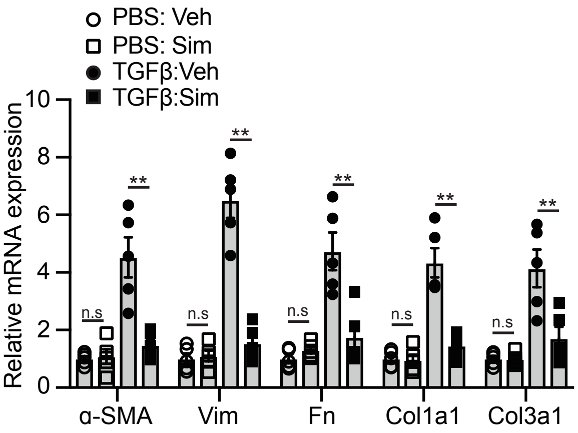

Supplementary Figure 6

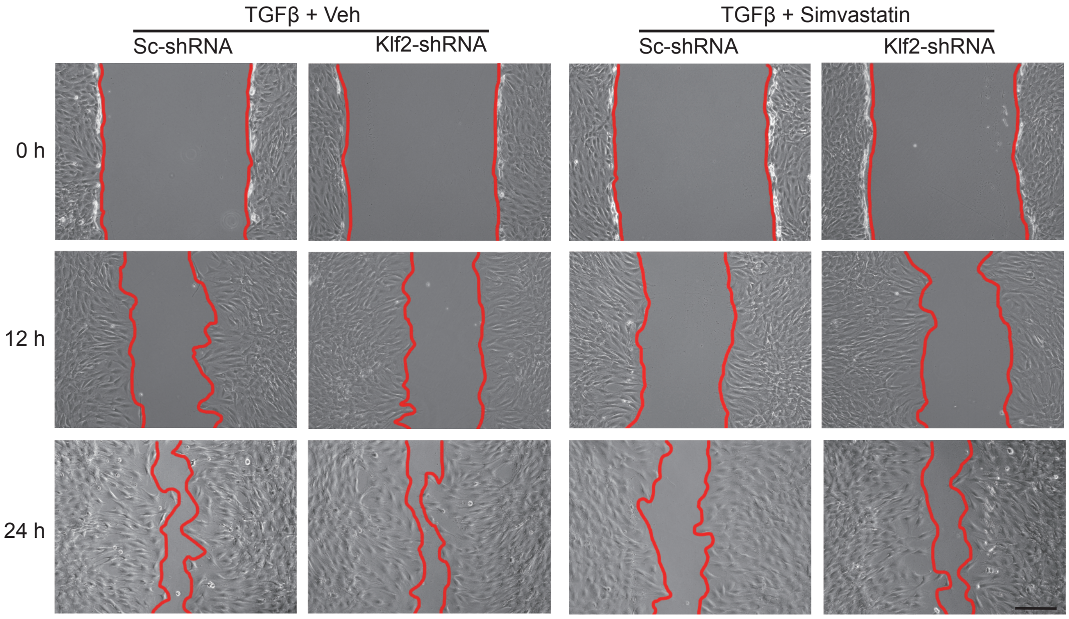

Supplementary Figure 7

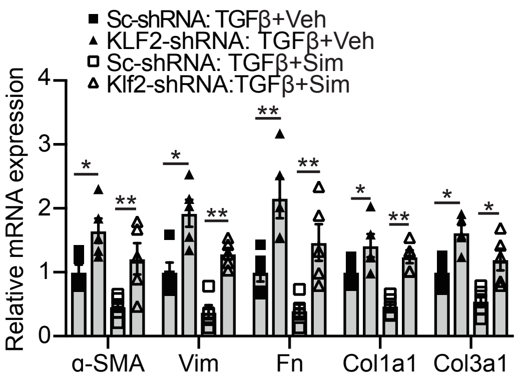

Supplementary Figure 8

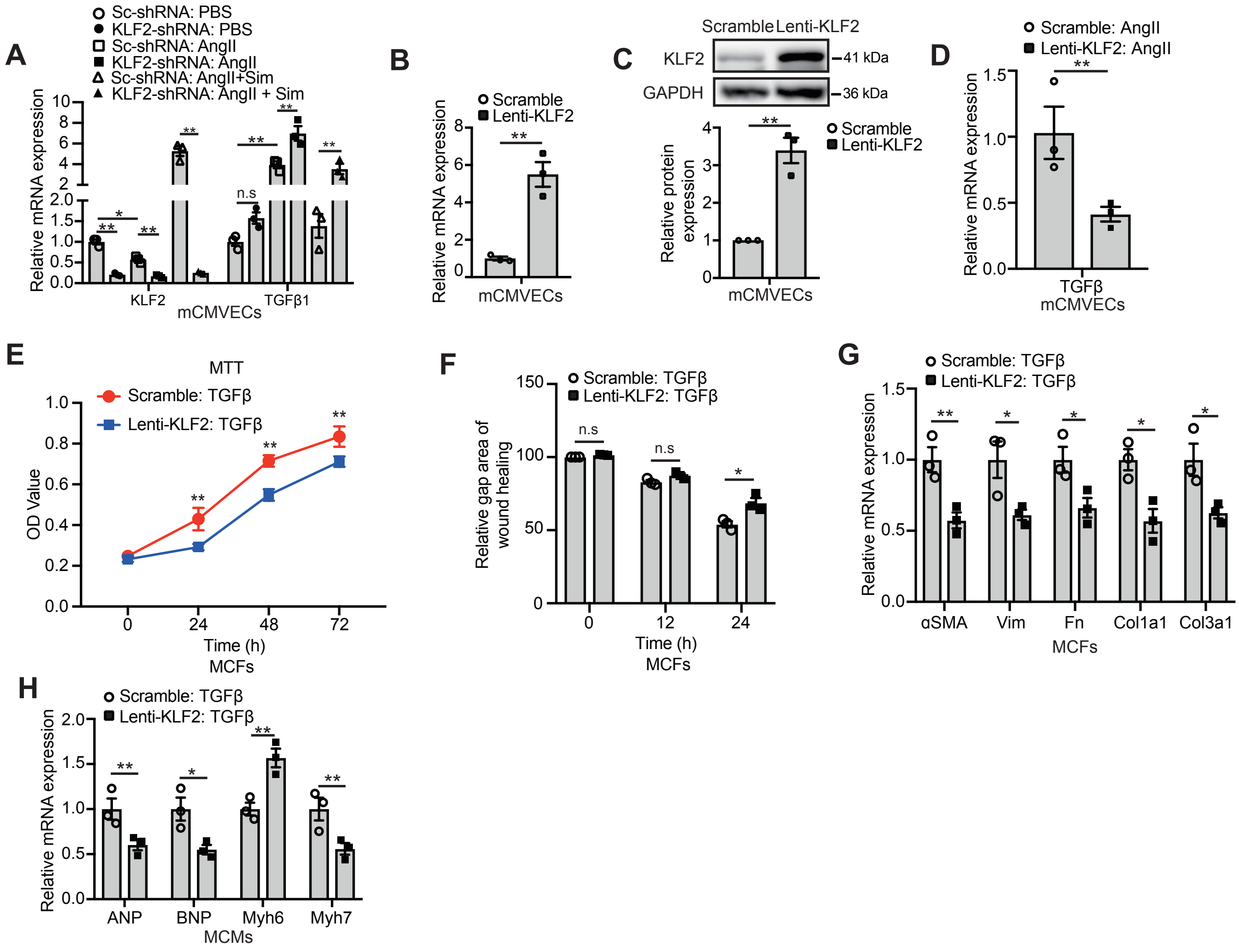

Supplementary Figure 9

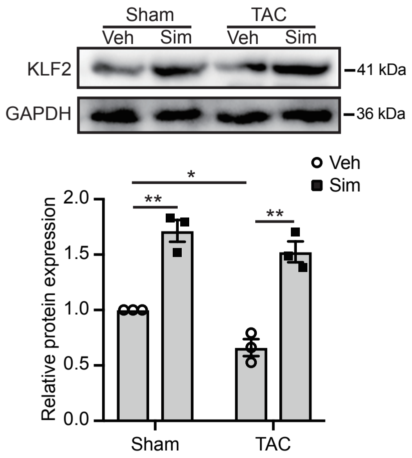

Supplementary Figure 10

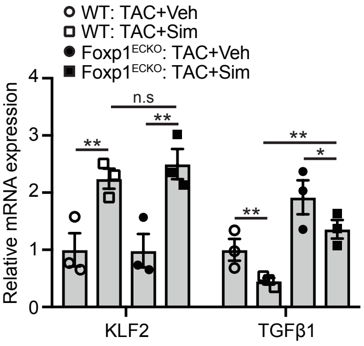

Supplement: Supplementary file 1 — Supplementary figures. [file thnov11p1609s1.pdf]
